# Postoperative analgesia of scalp nerve block with ropivacaine in pediatric craniotomy patients: a protocol for a prospective, randomized, placebo-controlled, double-blinded trial

**DOI:** 10.1186/s13063-020-04524-7

**Published:** 2020-06-26

**Authors:** Wei Xiong, Lu Li, Di Bao, Yaxin Wang, Yi Liang, Pengwei Lu, Di Zhang, Gaifen Liu, Lanxin Qiao, Na Zheng, Xu Jin

**Affiliations:** 1 Department of Anesthesiology, Beijing Tiantan Hospital, Capital Medical University, Beijing, 100070 China; 2 Department of Neurosurgery, Beijing Tiantan Hospital, Capital Medical University, Beijing, 100070 China; 3 Department of Neurology, Beijing Tiantan Hospital, Capital Medical University, Beijing, 100070 China; 4 China National Clinical Research Center for Neurological Diseases, Beijing, 100070 China

**Keywords:** Scalp nerve block, Ropivacaine, Children, Craniotomy, Postoperative analgesia, RCT, Protocol

## Abstract

**Background:**

Moderate-to-severe postoperative pain following craniotomy has a high incidence in pediatric patients. Such pain may cause agitation, intracranial hypertension, epileptic seizures, and postoperative hematoma, which affect morbidity and mortality. Although scalp nerve block (SNB) achieves satisfactory pain relief except for suboccipital mid-craniotomy in adults and ropivacaine is widely used as a long-acting peripheral nerve block agent in children, there are few studies of SNB with ropivacaine in pediatric patients undergoing craniotomy. In addition, the neurosurgery operation time is relatively long, but the duration of action of SNB is limited. It is generally believed that postoperative SNB is better than preoperative SNB for postoperative analgesia. However, considering the concept of preemptive analgesia, we believe that preoperative SNB may achieve a longer postoperative analgesia effect than we expected.

**Methods:**

This trial is a single-institution, prospective, randomized, controlled, double-blind study. A total of 180 children aged between 1 and 12 years who are undergoing elective craniotomy will be randomly allocated in a 1:1:1 ratio to three groups: group B (preoperative ropivacaine block group), group A (postoperative ropivacaine block group), and group N (nonblocking control group). This randomization will be stratified by age in two strata (1–6 years and 7–12 years). The primary outcome is the total consumption of sufentanil within 24 h after surgery. The secondary outcomes include assessment of pain scores, total consumption of sufentanil and emergency-remedy medicine consumption at observation points, the occurrence of postoperative complications, and the length of hospitalization after surgery.

**Discussion:**

This study is designed to explore the effect and feasibility of SNB with ropivacaine for postoperative analgesia in pediatric patients undergoing craniotomy. Further aims are to compare the effects of preoperative and postoperative SNB on postoperative analgesia in order to identify whether there is a preemptive analgesic effect and determine the better time to implement SNB in pediatric patients during craniotomy.

**Trial registration:**

Chinese Clinical Trial Registry ChiCTR1800017386. Registered on 27 July 2018.

## Background

Pain is defined as “an unpleasant sensory and emotional experience associated with actual or potential tissue damage or described in terms of such damage” according to the International Association for the Study of Pain (IASP) [1]. Fetal pain receptors develop at approximately the 26–30th weeks of gestation [2]. Pain can be perceived by individuals ranging from neonates to adolescents, and surgical trauma is an important cause of postoperative pain in children.

For a long time, craniotomy procedures have been considered to be associated with less pain than other surgical procedures; accordingly, fewer analgesic methods are implemented for craniotomies than for other surgical procedures [3]. Therefore, postoperative analgesia of neurosurgery patients has not received sufficient attention. Recently, studies showed that up to 80% of patients experience moderate-to-severe pain after craniotomy [4–10]. There are even fewer investigations on postoperative analgesia in pediatric neurosurgery patients. A study observed that 42% of 52 children undergoing craniotomy continued to experience moderate-to-severe pain within 72 h after surgery and without the use of analgesics [11].

The improper management of pain following neurosurgery may further result in severe consequences such as agitation, intracranial hypertension, epileptic seizures, and postoperative hematoma, which affect morbidity and mortality [12–15] and further lead to a more negative outcome for pediatric craniotomy patients. Moreover, according to long-term follow-up studies, postoperative pediatric pain has a substantial impact on children and may even cause long-term behavioral changes [16]. Currently, opioids are mainly used for the treatment of moderate and severe postoperative pain, but they have clear adverse effects; hence, researchers are exploring alternatives in which nerve block may be a solution for postoperative analgesia following craniotomy [17]. Scalp nerve block (SNB) achieves satisfactory results during either intraoperative awakening or postoperative analgesia, except for suboccipital mid-craniotomy in adults [14, 15, 18–21].

Ropivacaine, for long-action [22] and safety purposes, has become one of the most popular choices in children [23]. However, there are few studies of SNB with ropivacaine in pediatric patients undergoing craniotomy. In addition, the neurosurgical duration is relatively long, and the effective action time of SNB is limited. Postoperative pain is a time-dependent process that is considered to be the most severe within the first 24 h, gradually alleviates, and then disappears substantially at 72 h following surgery. The advantages of SNB following preoperative anesthesia include more stable hemodynamics, better postoperative analgesic effects, and less anesthetic consumption during surgery [24]. However, the duration of the action of SNB is limited. Craniotomy is known to be intricate, and subsequently, more time (typically > 4 h) will be occupied. Postoperative SNB is “timely and fresh” for postoperative analgesic withdrawal, and in theory, its analgesic effect is at least 4 h longer than the preoperative block effect. However, according to the mechanism of “pre-emptive analgesia,” if an analgesic agent is administered before the “tissue damage” occurs, the maximum results will be achieved with a small dose.

Because pain is a subjective indicator and some children, particularly infants, cannot properly express their pain, it is more difficult to evaluate pediatric pain than adult pain [25–27]. There is currently no ideal assessment scale for all pain types in children of all ages. To date, the Face, Legs, Activity, Crying, Consolability (FLACC) scale, Wong–Baker Faces Pain-rating Scale (FACES), and numerical rating scale (NRS) scores have effective assessment instructions for children. The former two scales, which are easily obtained by observers, can assess the pain intensity of children from 1 to 12 years of age. The NRS, which is rated according to the child’s report, can only be used for those aged 7–12 years.

### The Face, Legs, Activity, Crying, Consolability (FLACC) score [28]

The FLACC scale method is commonly used for pain assessment following pediatric surgery. In the FLACC scale, pain scores are obtained by medical staff based on the observed pediatric condition and contents of the quantification table. Each item is scored from 0 to 2 points. The sum of the content scores is the total score (from 0 to 10 points). A higher score indicates more severe pain. With the FLACC scale, physicians observe children for 1–15 min, and this tool is commonly used for the evaluation of postoperative pain in children aged 1–18 years and is the first evaluation method for hospitalized children.

### The Wong–Baker Faces Pain-rating Scale (FACES) facial pain assessment method [29]

This scale is primarily applicable to children between 1 and 18 years of age and for infants or children with communication difficulties. The score ranges from 0 to 10 points. However, it should be noted that children may lose their “smiley face” because of fear, hunger, or other stressors. Therefore, the possible influences of these factors should be excluded from the pain assessment when using this scale.

### The numerical rating scale (NRS)

This scale ranges from 0 to 10 points. A score of 0 indicates no pain, scores of 1–3 indicate slight pain tolerable and does not affect rest, scores of 4–6 indicates that the pain affects sleep but is tolerable, and scores of 7–10 indicate that the pain is intolerable and affects appetite and sleep.

## Methods

### Trial design and study setting

This trial is a single-institution, prospective, randomized, controlled, double-blind study that is designed to explore the effect and feasibility of SNB with ropivacaine for postoperative analgesia in pediatric patients undergoing craniotomy. We further compared the effect of preoperative and postoperative SNB on postoperative analgesia in pediatric craniotomy. From July 2018 to October 2020, pediatric patients aged 1–12 years presenting for elective craniotomies will be recruited from Beijing Tiantan Hospital, Capital Medical University.

**Fig. 1:**
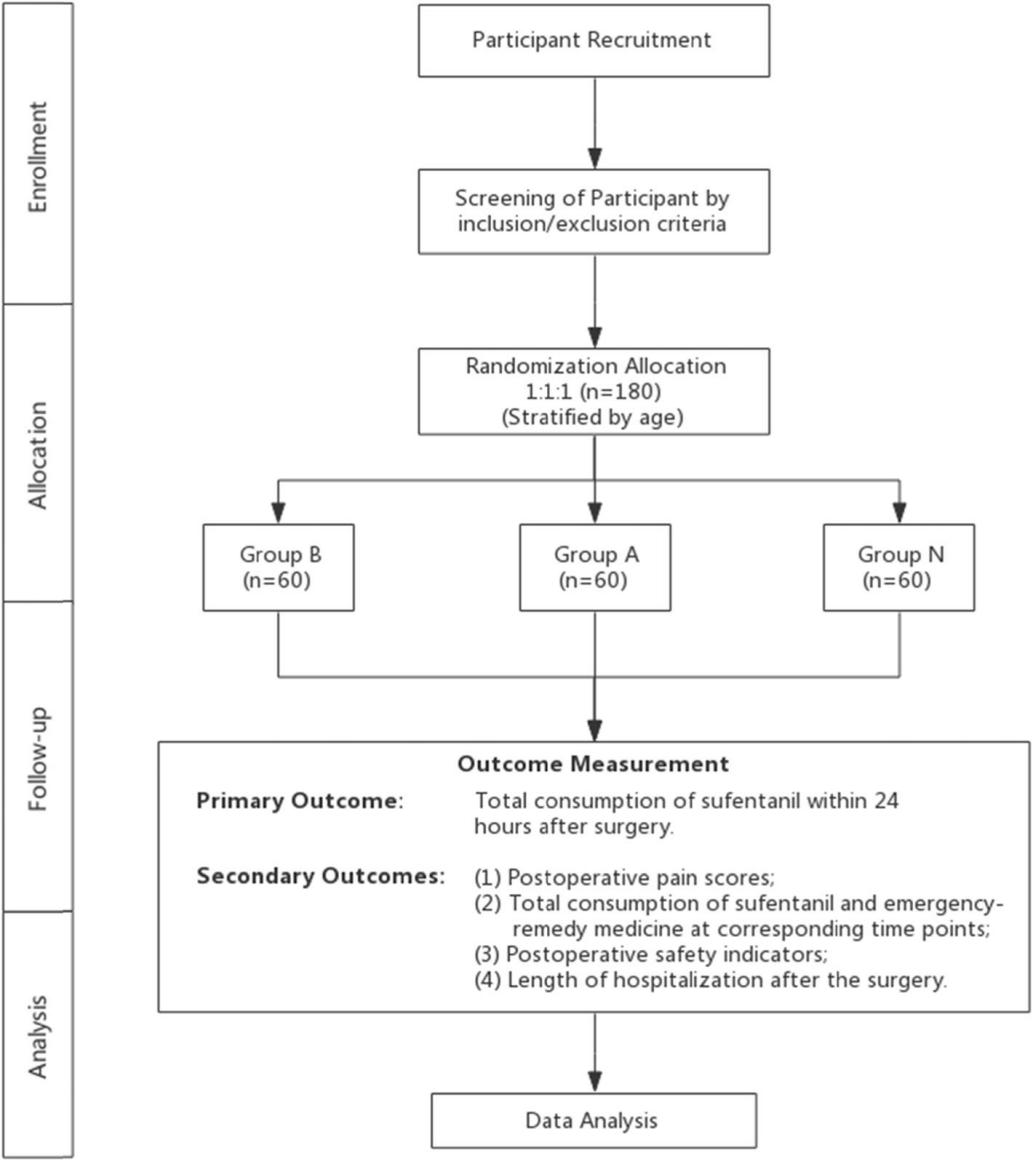
Consolidated standards of reporting trials (CONSORT) flow diagram. Group B: preoperative ropivacaine block group; group A: postoperative nerve block group; group N: nonblocking control group

The patient flow diagram of the study is presented in Fig. 1, and the study schedule is shown in Table 1.

**Table 1:** The schedule of enrollment, interventions, and assessments

		Study period									
		Enrollment	Allocation	Postallocation							
Timepoint		Day − 1	Day 0	After induction, before surgery	After surgery, before extubation	1 h	2 h	4 h	24 h	48 h	Discharge time
Enrollment											
Eligibility screen		X									
Informed consent		X									
Randomization			X								
Allocation			X								
Interventions											
[Group B]	Blocking			X							
	Dressing			X	X						
[Group A]	Blocking				X						
	Dressing			X	X						
[Group N]	Blocking										
	Dressing			X	X						
Assessment											
[Analgesics and remedies]						X	X	X	※	X	
[Pain scores]			X			X	X	X	X	X	
[Complications]						X	X	X	X	X	
[Serious adverse events]						X	X	X	X	X	
[Length of hospitalization]											X

※ primary outcome

### Eligibility

Trial investigators will identify consecutive eligible patients based on the inclusion and exclusion criteria the day before the operation. When patients meet the eligibility criteria, an investigator will explain the study protocol and relevant information to the authorized surrogates and participants. Informed written consent will be obtained from the authorized surrogates and participants (over 7 years) themselves, and then they will be instructed how to use the electronic analgesia pump.

Inclusion criteria:

1. Age of 1–12 years
2. American Society of Anesthesiologists (ASA) physical status of I–III
3. Craniotomy for tumor resection includes frontal, temporal, parietal, and bifrontal regions.

Exclusion criteria:

1. Children with severe diseases or cardiac insufficiency
2. Presence of airway abnormalities and reactive airway diseases
3. Children who cannot be weaned from endotracheal intubation following surgery
4. Children with abnormal liver and kidney function test results (when alanine aminotransferase, aspartate aminotransferase, blood urea nitrogen, and creatinine levels are higher than or equal to 1.5 times the reference value)
5. Children who have participated in other clinical trials
6. Children whose authorized surrogates are unable or unwilling to provide informed consent or poor compliance
7. Children who have mental illness or use antipsychotic drugs for treatment
8. Posterior fossa craniotomy for tumor resection

### Allocation and randomization

After meeting the eligibility criteria and signing the informed consent to participate in the study, 180 patients will be randomly allocated in a 1:1:1 ratio to three groups: group B (preoperative ropivacaine block group), group A (postoperative ropivacaine block group), and group N (nonblocking control group), stratified by age into two strata (aged 1–6 years and aged 7–12 years). The allocation schedule will be made with a computer random number list generated by Stata software version 15.1.

### Intervention

The corresponding SNB schemes of the three groups will be the following:

- *Group B*: Preoperative SNB group (ropivacaine hydrochloride injection: Naropina®, AstraZeneca AB, Sweden): SNB will be performed after general anesthesia and endotracheal intubation.
- *Group A*: Postoperative SNB group: SNB will be performed after the end of the operation, and the effects of analgesics and sedatives will not wane.
- *Group N*: nonblocking control group: This control group will not undergo SNB.

SNB will be performed exclusively by a well-trained anesthesiologist with two age-appropriate concentrations of ropivacaine: 0.3% in children aged 1–6 years and 0.5% in children aged 7–12 years.

The site of SNB will be selected according to the craniotomy region. Dosage: supraorbital nerve (1–2 ml), auriculotemporal nerve (1–2 ml), zygomatic temporal nerve (1–2 ml), greater occipital nerve (2–3 ml), and lesser occipital nerve (2–3 ml). An experienced anesthesiologist will determine the injection points according to the surgical incision site. The total volume will not exceed 5 ml.

The independent anesthesiologist performing the SNB will not be an investigator in charge of the operation and will not visit patients before or after the operation. The anesthesiologist will enter the operating room twice, the first time is after endotracheal intubation prior to surgery, and the second time is after suturing the scalp. He/she will confidentially perform or not perform SNB according to the allocation plan and cover the dressing at the corresponding portion to conceal the allocation. Due to ethical care standards for children, normal saline will not be injected, and only a dressing will be covered in group N.

### Anesthesia and analgesia

On arrival to the operating room, standard monitoring will be established (noninvasive blood pressure (BP), heart rate (HR), pulse oximetry saturation (SpO_2_), invasive arterial pressure, end-tidal carbon dioxide partial pressure (P_ET_CO_2_), anesthesia gas monitoring). Midazolam (0.025–0.075 mg kg^−1^) and methylprednisolone (0.1–0.2 mg kg^−1^) will be received intravenously before anesthesia induction. A dose of 0.5 mg kg^−1^ midazolam will be administered orally to children in whom intravenous access cannot be established due to crying and agitation. Peripheral vascular access will be obtained after sufficient sedation, and anesthesia will be induced with 0.5 μg kg^−1^ sufentanil, 2–3 mg kg^−1^ propofol, and 0.1–0.2 mg kg^−1^ cis-atracurium or 0.4–0.6 mg kg^−1^ rocuronium. After intubation, mechanical ventilation will be in a volume-controlled mode with a tidal volume set at 8–10 ml kg^−1^, and the respiratory rate will be set at 14–20 breaths per minute. Total intravenous infusion anesthesia will be maintained with remifentanil and propofol (8 mg kg^−1^ h^−1^). The initial dose of remifentanil will be 0.1–0.2 μg kg^−1^ min^−1^, adjusted for analgesic needs during operation. The infusion of propofol and remifentanil will be stopped after surgery. Tramadol 1 mg kg^−1^ will be administered half an hour before the end of surgery. The mean arterial pressure (MAP) and HR will be maintained within 30% of the baseline values, and if exceeding this range, corresponding treatment will be taken. No other additional analgesics will be administered during surgery. The total dosage of analgesics and anesthetics will be estimated after surgery. Local infiltration of the head incision will not be performed before surgery. During the operation, the child’s temperature will be monitored and maintained at 35–38 °C. After extubation, patients will be transferred to the postoperative care unit (PACU) or ICU.

At the time of remifentanil halt, an electronic analgesia pump (Apona® electronic infusion pump ZZB-I-150, APON Medical Technology Co., Ltd., Jiangsu, China) will be routinely applied. The electronic analgesia pump includes a bag loaded with 2 μg kg^−1^ sufentanil and 0.3 mg kg^−1^ ondansetron diluted in 100 ml of normal saline. There will be no background infusion doses. Press the electronic analgesia pump when the FLACC pain score is > 4. If the patient is still in the operating room, the anesthesiologist can determine whether to press the button. In the PACU, ICU, or ward, children aged 1–6 will be placed in a nurse-controlled intravenous analgesia mode. Children aged 7–12 will have their analgesia administered by a nurse until they are able to operate the button of the electronic analgesia pump. The electronic analgesia pump will provide a bolus dose of only a 2-ml infusion each time and a lock-out period of 30 min. If the FLACC pain score is > 5 and the FACES facial pain score is > 6, the remedial procedure will be initiated.

Administration of analgesics and remedies: Children who still show insufficient analgesia or discomfort after the operation and require analgesics will be administered acetaminophen at 15 mg kg^−1^ orally. Information on whether additional analgesics will be needed within 3 days after surgery as well as on the drug dosage and frequency and the usage of the postoperative electronic analgesia pump (including total consumption of analgesics and the number of effective compressions and invalid compressions) will be recorded.

### Outcomes measurement

Primary outcome: Total consumption of sufentanil within 24 h after surgery.

Secondary outcomes:

1. Assessment of postoperative pain scores at 1, 2, 4, 24, and 48 h after surgery: children aged 1–6 years: due to the special characteristics of children after neurosurgery and of preschoolers who cannot properly express their pain with language, two different scoring methods will be used—the FLACC scale and the FACES facial pain score. Children aged 7–12 years can use NRS for self-assessment in addition to the above two methods.
2. Total consumption of sufentanil within 1, 2, 4, and 48 h after surgery and emergency-remedy medicine consumption at 1, 2, 4, 24, and 48 h after surgery.
3. Postoperative safety indicators: complications including agitation, postoperative nausea and vomiting (PONV), bleeding, infection, hypoxemia, respiratory depression, neurological impairment at 1, 2, 4, 24, and 48 h after surgery, and serious adverse events including disability and death.
4. Length of hospitalization after the surgery.

### Criteria for discontinuing or modifying the allocated interventions

There will be no special criteria for modifying allocated interventions, but there are conditions under which to terminate the study: (1) unexpected postoperative continuation of intubation; (2) serious adverse events during the operation, such as acute massive hemorrhage, malignant fever, and shock; (3) unplanned reoperation within 24 h after surgery; (4) serious, adverse surgically related events/reactions occurring within 24 h after the operation, such as postcraniotomy hematoma, unconsciousness, coma, and cardiac arrest; and (5) serious adverse events/reactions related to the medicines in electronic analgesia pumps within 24 h after surgery.

### Strategies to improve the adherence to intervention protocols

The anesthesiologist who performs the SNB is well skilled and will be trained to perform the standard intervention method. The anesthesiologists in charge will be blinded when he/she performs anesthesia and records the intraoperative data. The investigator group will be blinded and well trained to perform preoperative recruitment and postoperative follow-up. Nurses in the PACU, ICU, or ward will be trained on how to use the electronic analgesia pumps and when to press the button of the electronic analgesia pump (nurse-controlled intravenous analgesia). In addition, participants and authorized surrogates will also explain the usage of electronic analgesia pump. Any deviations and missing data will be recorded.

### Blinding

After the patient’s enrollment, the independent anesthesiologist who performs the SNB will obtain the allocation randomized schedule from computer software operation. The allocation information of participants who are recruited and randomized will be placed in a sealed envelope and stored individually. Therefore, the anesthesiologist who performs the SNB will be the only person who knows the allocation schedule. This procedure ensures that both investigators and participants will not know the intervention.

Patients, anesthesiologists in charge of operation, and investigators who are responsible for enrolling and observing the primary and secondary outcomes will not know whether the children will receive blocking. The blinding will be discontinued after all data collection is completed. Furthermore, serious life-threatening adverse events leading to prolonged hospital stay or death will be reported to the principal investigator (PI), and the blinding will be broken following consultation with the PI if necessary.

### Data collection and management

Investigators will explain the benefits of participating in the trial to patients and their authorized surrogates before surgery. After the operation, a specific blinded investigator will conduct follow-up in the ward at 1, 2, 4, 24, and 48 h. Participants who are unable to complete the assessment of the primary outcome will be excluded in situations such as death or discharge in 24 h. Cases with other deviations and deficiencies except for the aforementioned conditions will be retained. Outcome investigators will receive training on all outcome measures. Nurses, children, and their authorized surrogates will be trained to use the electronic analgesia pump. All electronic analgesia pumps will be calibrated by a researcher according to uniform standards. The anesthesiologists in charge will record the patients’ intraoperative data. The relevant data of the electronic analgesia pumps will be recorded by an electronic memory system. The data are accessible to investigators who are blinded to the trial. A blinded investigator will collect other data on secondary outcomes, such as pain scores, postoperative complications, and length of hospitalization after surgery. The data managers will use double data entry to enter data into the EpiData database. An inspector will examine the data, create records, and revise these records as necessary.

Each participant will have a unique study identifier, and their data will be recorded by an independent data manager. The data will be electronically stored in the EpiData database and undisclosed to other researchers until the study is completed. The final dataset will be handed over to statistical analysts for statistical analysis. Regular data checks and double data entry will be applied to promote data quality.

### Confidentiality

Before the trial, all personal information will be obtained only by the PI who has signed a confidential disclosure agreement. After randomization, during and after the trial, unique study identifiers will be assigned to each participant to reduce the risks of accidental disclosure of identifiable information. To protect confidentiality, paper information will be stored in a locked cabinet inside the locked research office. The electronic information will also be kept in a password-protected electronic database. The specific privilege assignments to access the database to acquire personal information will be limited by the role of the assignees in the trial. Only code numbers will appear on any data and documents used for evaluation or statistical analyses.

### Sample size

The primary outcome in this study is the total consumption of sufentanil within 24 h after surgery. A decrease of 20% will be considered a minimal clinically important difference. According to Maxwell’s article [30] and our experience, 54 subjects per group will be necessary with a two-sided *α* level of 0.017 (0.05/3) and 80% power. Considering a 10% rate of loss to follow-up, it would be necessary to include 60 participants per group (total: 180 participants). The sample size calculation is performed using PASS 15.0.

### Statistical analysis

Statistical analyses will be performed using SPSS 23.0 software. All measured data will be reported as the mean ± standard deviation ($\overline{x}$ ± s), interquartile range (IQR, 25–75% percentile), or number (%). For normally distributed and equal-variance data, statistical analyses of categorical variables will be carried out using the *t* test or analysis of variance (ANOVA). For abnormally distributed and unequal variance data, statistical analyses of categorical variables will be carried out using a nonparametric test as appropriate. A chi-square test and Fisher’s exact test will be used to compare proportions. For the primary outcome, we will first use ANOVA to compare whether there are differences among the three groups. If there are differences, further multiple comparisons will be performed. The results of the FLACC scores, FACES scores, and NRS scores will be analyzed by repeated measurements for general linear models and multivariate analysis. We will also perform the subgroup analysis based on different ages. We will exclude patients who reject the intervention and whose primary data are missing. Statistical significance will be defined as a *P* value < 0.05.

All randomized participants with informed consent will be analyzed. If unintended missing data related to the primary outcome account for more than 10%, this will be handled with multiple imputation. Analyses will be performed according to the intention-to-treat principle.

### Dissemination

The study protocol has been registered and is available on the Chinese Trial Registry website (registered in ChiCTR.org with the identifier ChiCTR1800017386). The results will be disseminated to all participants, researchers, and healthcare providers through study summary documents, courses, presentations, and the Internet. The datasets analyzed during the current study are available from the corresponding author on reasonable request.

## Discussion

All pediatric patients who undergo craniotomy require analgesic medications for postoperative analgesia. SNB is a simple strategy that can relieve postoperative pain. In this trial, we hypothesize that ropivacaine, which is the SNB administered in pediatric patients undergoing neurosurgery, will decrease the requirement for sufentanil and other pain medications as well as reduce postoperative pain scores. The second aim is to determine whether preoperative block or postoperative block is more suitable for postoperative pain control. This study will be a prospective, randomized, controlled, double-blind study. Participants are randomized into three groups: preoperative SNB group, postoperative SNB group, and nonblocking control group, who will be followed for 48 h postoperatively and then the day of discharge. We have set the same standardized anesthesia protocol to avoid sources of bias and will use the FACES facial pain assessment method based on the FLACC scale to improve the accuracy of pain measurement. The study will be carried out at Beijing Tiantan Hospital, Capital Medical University (which has the highest rank for neurosurgery in China). This investigation is a single-center study, which may bias our results. However, the results of this study may promote the development of postoperative analgesia and popularize the optimal pain management of children.

### Trial status

The version number of this Clinical Research Program is V2.0, and the version date is 20180830. The first patient was recruited on September 13, 2018. Recruitment is expected to end in October 2020.

## Supplementary information

**Additional file 1.**

## Data Availability

The material of this study will be conserved in a secure repository at the Anesthesiology Department, Beijing Tiantan Hospital, Capital Medical University. Datasets will be available from the PI upon reasonable request.

## Abbreviations

SNB: Scalp nerve block
RCT: Randomized controlled trial
ASA: American Society of Anesthesiologists
IASP: International Association for the Study of Pain
FLACC score: Face, Legs, Activity, Crying, and Consolability score
FACES: Wong-Baker Faces Pain-rating Scale
NRS: Numerical rating scale
BMI: Body mass index
P_ET_CO_2_: End-tidal carbon dioxide
SpO_2_: Pulse oximetry saturation
MAP: Mean arterial pressure
HR: Heart rate
SD: Standard deviation
IQR: Median and interquartile range
AE: Adverse effect
PONV: Postoperative nausea and vomiting
IRB: Institutional review board
CC: Coordination center
PI: Principal investigator
TSC: Trial steering committee
