# Supplementary material for: Postoperative analgesia of scalp nerve block with ropivacaine in pediatric craniotomy patients: a protocol for a prospective, randomized, placebo-controlled, double-blinded trial
Source: Trials. 2020 Jun 26;21:580. doi: 10.1186/s13063-020-04524-7 (PMC7318534; doi:10.1186/s13063-020-04524-7)
Supplement: Supplementary file 1 — Additional file 1. [file 13063_2020_4524_MOESM1_ESM.zip › reference 10R2.pdf]

Pain after Major Craniotomy in a University Hospital: A Prospective Cohort Study

Sirilak Suksompong MD, MSc*,
Nophanan Chaikittisilpa MD*, Tadrawee Rutchadawong MD*,
Ekawut Chankaew MD**, Benno von Bormann MD*

** Department of Anesthesiology, Faculty of Medicine, Siriraj Hospital, Mahidol University, Bangkok, Thailand*
*** Division of Neurosurgery, Department of Surgery, Faculty of Medicine, Siriraj Hospital, Mahidol University, Bangkok, Thailand*

Background: *Pain after major craniotomy has been believed to be less severe than the other operations.*
Objective: *To determine the incidence and risk factors of moderate to severe pain after major craniotomy.*
Material and Method: *This is a prospective observational study in a neurosurgical intensive care unit and wards of a university, tertiary hospital. After institutional IRB approval, patients undergoing major craniotomy during May 2011-August 2012 were interviewed preoperatively and 24 and 48 hours postoperatively. Demographic data, preoperative anxiety scores, operative data and postoperative pain characteristics were recorded.*
Results: *Two hundred and eighty patients completed the study. The incidence of moderate to severe pain was 75%. Mean pain score during 24 and 48 hours were 5.5±2.7 and 3.5±2.6, respectively. Univariate analysis identified age under 45 years and perioperative steroid therapy as predictors of moderate to severe postoperative pain. Using multivariate analysis, only age under 45 years was a significant risk factor. Patients' satisfaction scores were good in both mild and moderate to severe pain groups (9.49±1.08 and 8.37±1.76). During postoperative period, almost all of the patients received intravenous opioid and oral acetaminophen for pain treatment. No respiratory depression occurred, but postoperative nausea and vomiting occurred in 51.7% and pruritus in 23.6%.*
Conclusion: *Incidence of pain after craniotomy was high especially in younger age group, which is not in accordance with all similar reports. However, we believe pain management after major craniotomy in our hospital requires improvement.*

Keywords: *Postoperative pain, Craniotomy, Incidence, Risk factors*

J Med Assoc Thai 2016; 99 (5): 539-48
Full text. e-Journal: *http://www.jmatonline.com*

Pain after craniotomy has long been believed to be mild to moderate degree, thus bearable. Dunbar et al[1] had demonstrated that most of the patients undergoing major intracranial surgery had less pain than those with open fixation of mandible or maxilla or lumbar laminectomy. However, they also found that patients who underwent frontal craniotomy had moderate to severe pain requiring aggressive pain therapy. Gottschalk et al[2] had shown that 69% and 48% of the patients with major craniotomy had moderate to severe pain on the first and second postoperative day. De Benedettis et al[3] showed that in patients after craniotomy about 42% had moderate to severe pain during the first 48 postoperative hours. The survey by Stoneham et al[4] demonstrated that the most common analgesic used was intramuscular codeine phosphate or dihydrocodeine. Finally, more than 50% neuroanesthesiologists judged postoperative analgesia to be inadequate. Insufficient postoperative pain relief may result from therapists exercising restraint in giving potent opioids like morphine to avoid both respiratory depression and masking the reaction of pupils. However, unrelieved postoperative pain might lead to hypertension with consequences much more dangerous, such as cerebral hemorrhage[5] and development of chronic pain[6]. Nair et al[7] had found that three out of six patients with moderate pain at 24 hour postoperatively developed meningitis and bleeding.

Pain after major craniotomy depends on several factors such as surgical site (supratentorial or in fratentorial), gender, age and anxiety. However, some of these risk factors such as surgical site are debatable. Thibault et al[8] found frontal craniotomy to

Correspondence to:
Suksompong S, Department of Anesthesiology, Faculty of Medicine, Siriraj Hospital, Mahidol University, 2 Wanglang Road, Bangkoknoi, Bangkok 10700, Thailand.
Phone: +66-2-4197995, Fax: +66-2-4113256
E-mail: sirilak.suk@mahidol.ac.th

be associated with less pain and lower postoperative analgesic requirement when compared to posterior fossa craniotomy. Gottschalk et al[2] had shown that patients with infratentorial approach experienced more pain than those with supratentorial approach, whereas Irefin et al[9] could not demonstrate a relation between pain and craniotomy site.

The primary objective of our study is to evaluate the incidence of moderate to severe pain following major craniotomy in our hospital. We also evaluate the risk factors of moderate to severe pain, the quality of pain management and patients' satisfaction with it.

Material and Method

This prospective cohort study was approved by the Siriraj Institutional review board. Trial registration was made at ClinicalTrials.gov, ID: NCT01502540. Patients aged more than 18 years old undergoing elective major craniotomy between May 2011 and August 2012 were included. Exclusion criteria were inability to communicate or rating of pain score, psychiatric problems, history of drug abuse and emergency surgery. After informed consent was obtained, each patient was informed, evaluated and questioned by one of the authors. The day before surgery, they were taught how to use numeric rating scale score (NRS 0-10) for postoperative pain assessment, preexisting pain and treatment, expected postoperative pain score, and preoperative anxiety using the Amsterdam Preoperative Anxiety and Informative Scale (APAIS)[10]. In addition nine short questions were asked regarding specific factors in preoperative anxiety which were modified from the study of Perks et al[11]. The APAIS composes of six questions, which are divided into anxiety (APAISa), and knowledge (APAISk) components. The patients were assigned to high level of anxiety if APAIS a was $\geq$10[11]. They were then visited twice on the first and second postoperative day by one of the authors to ask the patient to rate the maximal NRS score during 24 and 48 hours. The patients were also evaluated about side effects and satisfaction of pain management. The factors that were included in analysis as predictors of moderate to severe pain were gender, age, ASA classification, educational level, craniotomy site, smoking, preexisting pain and duration, APAIS, perioperative steroid usage, operative time, intraoperative fentanyl dosage, and postoperative wound infiltration with local anesthetic agents.

Pain was considered as mild (NRS 0-3), moderate (NRS 4-7) and severe (NRS 8-10). Opioid and non-opioid analgesic consumption were recorded. Fentanyl 10 mcg, meperidine 10 mg and parecoxib 10 mg were considered equipotent to morphine 1 mg[12,13]. We also evaluated the effectiveness of our routine acute postoperative pain service in our hospital. We have a policy to monitor NRS score every hour for the first 12 hours, every 2 hours for the next 12 hours and every 4 hours for the next 24 hours after surgery. We have graded the acute pain management into 4 categories: 1) reasonable pain management when NRS score is $\geq$4 and under pain medication or NRS score <4 with or without treatment or asleep, 2) fair management when analgesics were applied without NRS recording, 3) poor pain management when there was no treatment even in patients with NRS $\geq$4, and 4) no pain assessment.

Sample size (300 patients) was calculated to detect an accepted variation of 0.055 with a type I error of 0.05 and 10% dropout. The incidence of moderate to severe pain after major craniotomy is 69%, based on the previous study[2]. Data were analyzed using SPSS version 16.0 software (SPSS, Inc., Chicago, IL, USA). Continuous data such as age, weight, NRS, operative time, and anesthetic time are presented as mean and standard deviation. Categorical data such as gender and ASA physical status are presented as numbers and percentages. Univariate analyses were performed using the Chi-squared test or Fisher's exact test, as appropriate. Significant variables (p<0.25) were entered into multivariable logistic regression analysis, using the backward stepwise method with combined mortality and morbidity as the dependent variable and significant set at the 0.05 level. A multiple logistic regression analysis of risk of moderate to severe pain was developed. A p-value <0.05 was considered statistically significant.

Results

There were 302 patients enrolled in the study. Twenty-two patients were excluded due to inability to rate NRS pain score during the first postoperative day (POD). A total number of 280 patients (83 male and 197 female) remained for data analysis. However five patients were unable to rate NRS pain score on the second POD. Demographic and perioperative data are summarized in Table 1 and 2. Most of our patients were female, ASA I-II, and underwent supratentorial approach.

The incidence of moderate to severe pain during the first 48 hours postoperatively was 75% (Fig. 1). Maximum pain score during 24 and 48 hours

Table 1. Demographic and preoperative data

Variables	Mean ± SD or n (%) n = 280
Gender	
Male	83 (29.6)
Age (years)	
<45	98 (35.0)
45-60	139 (49.6)
>60	43 (15.4)
ASA Class I-II	262 (93.5)
ASA Class III	18 (6.5)
Education	
Below bachelor degree	199 (71.1)
Bachelor degree and above	81 (28.9)
Smoking	
Never	217 (77.5)
Ex-smoker	48 (17.1)
Current smoker	15 (5.4)
Preexisting pain	
No	169 (60.4)
≤3 months duration	47 (16.8)
>3 months duration	64 (22.9)
APAISa; high level of anxiety (≥10)	54 (19.3)
APAISk; need for information (≥3)	157 (56.1)
Preoperative expectation of postoperative pain score (NRS 0-10)	
Mild (0-3)	71 (25.4)
Moderate to severe (4-10)	209 (74.6)

NRS = numeric rating scale score, ASA = American society of Anesthesiology, APAISa = Amsterdam preoperative anxiety and informative scale anxiety component, APAISk = Amsterdam preoperative anxiety and informative scale knowledge component

Table 2. Perioperative data

Variables	Mean ± SD or n (%) n = 280
Diagnosis	
Brain tumor	251 (89.6)
Vascular surgery	10 (3.6)
Others	19 (7.2)
Craniotomy site	
Supratentorial	205 (73.2)
Infratentorial	75 (26.7)
Patients position during operation	
Supine	190 (67.9)
Prone	32 (11.4)
Parkbench and lateral	42 (15.0)
Sitting	16 (5.7)
Perioperative steroids	
Yes	184 (65.7)
Operative time (hours)	
≤4	164 (58.6)
Intraoperative fentanyl (mcg/kg/hour)	
≤1	240 (85.7)
Postoperative pain perception compare to preoperative expectation	
Less	179 (63.9)
Same	64 (22.9)
Worse	32 (11.4)
0-24 hour morphine-equivalent dosage consumption (mg)	8.50±4.3
24-48 hour morphine-equivalent dosage consumption (mg)	2.70±2.6
Total morphine-equivalent dosage consumption (mg)	9.30±5.3
0-24 hour acetaminophen consumption (g)	1.25±0.55
24-48 hour acetaminophen consumption (g)	1.91±0.97

was on average 5.52±2.70 and 3.49±2.60, respectively, which was statistically significant (p<0.0001). There were 24 patients who had more pain on the second than the first postoperative day, most of them (83.3%) being in the group with moderate to severe pain. The characteristics of pain were pulsatile or pounding headache (76.4%), steady pain (22.9%); while the rest could not describe the characteristic of pain.

Most patients with mild pain (72.9%) experienced less pain than preoperatively expected, which was 62.4% in patients with moderate to severe pain (p = 0.009). The first postoperative day satisfaction scores for the pain management were 9.5±1.1 in patients with mild and 8.4±1.8 in patients with moderate to severe pain (p<0.0001). The second postoperative day satisfaction scores for the pain management were 9.8±0.8 in patients with mild and 8.9±1.5 in patients with moderate to severe pain (p<0.0001).

Predictors of moderate to severe pain identified using univariate analysis are patients' age <45 years and perioperative steroid administration (Table 3). However, high level of anxiety was also nearly significant (p = 0.054). The results were partly confirmed by multivariable logistic model, which showed that only age under 45 years was a significant predictor of relevant postoperative pain.

Almost all patients (98.9%) received morphine for postoperative pain relief, 3.9% got fentanyl, 1.8% meperidine and 17.9% parecoxib. The mean total morphine equivalent consumption during the first 24

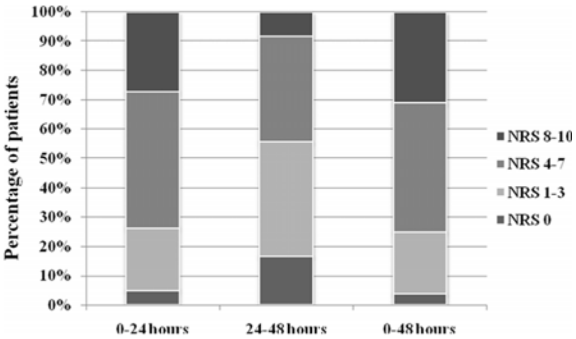

Fig. 1 Distribution of maximal numeric rating scale (NRS) score for pain assessment at 0-24, 24-48 and 0-48 hours.

hours postoperative was 6.2±3.6 mg and 9.3±4.2 mg in patients with mild and moderate to severe pain, respectively ($p<0.0001$). Postoperative oral acetaminophen usages were 1.25±0.55 and 1.90±0.97 grams for the first 24 hours and 24-48 hours, respectively (Table 4).

During the immediate postoperative period, routine pain assessment by the nurse was low (20% at the eleventh hour), with an increasing tendency afterwards (78% at the forty-eighth hour) (Fig. 2). Within that scoring (0-48 hour) only a small group of patients was categorized as receiving bad pain management. The percentage of patients with good pain management was higher on the second postoperative day compared to day one.

Postoperative nausea and vomiting (PONV) occurred in 51.7%, pruritus in 23.6%; no respiratory depression could be observed. There were 26.4% of patients with high anxiety scores (APAISa), 85.1% of them being female ($p = 0.001$). Patients concerns were mainly related to the result of the operation (65%), the possibility of physical/mental harm (62.1%), not knowing what is going to happen (54.3%) and postoperative pain (48.9%).

Discussion

The incidence of moderate to severe pain (75%) within 48 hours we observed in our study is in accordance with other investigations. Gottschalk et al[2] reported an incidence of postoperative pain in the first 24 hours after major craniotomy of 40-87%, results which are similar to others[7,14,15]. The maximum NRS score during the first postoperative day in our study was 5.5 which is quite high compared to others: less than three[14] and equal to five[2]. In the cited studies, the majority of patients had lower pain scores and analgesic consumption from the 1st to 2nd postoperative day[2,14]. However, in our study a small group of patients experienced increasing pain on the second day. This may be due to in adequate pain treatment formerly.

We demonstrated that age under 45 years being the only risk factor for moderate to severe pain after major craniotomy, which is consistent with other studies. Mordhorst et al[14] also demonstrated that the probability of experiencing pain after craniotomy is reduced by 3% for each additional year of life. Similar to this group[14] we did not find gender to be a significant risk factor for moderate to severe pain. Though more female experienced moderate to severe pain than male patients. Contrary to these findings, Gottschalk demonstrated female patients being more likely to experience postoperative pain after intracranial surgery[2].

Whether craniotomy site is a predisposing factor for postoperative pain is still a matter of debate. In our study, the surgical approach, supratentorial or in fratentorial, had no influence on the incidence of moderate to severe pain, which is in accordance with other studies[9,14], whereas Gottschalk et al[2] found the in fratentorial approach compared to supratentorial approach leading to increased postoperative pain. Additionally, Thibault et al[8] had demonstrated frontal craniotomy resulting in less postoperative pain compared to craniotomy at fronto-temporal, temporal, parietal, occipital and posterior fossa sites. They also demonstrated that patients with frontal craniotomy need fewer postoperative analgesics than posterior fossa craniotomy, which is in accordance to others with similar results, including patients requiring more postoperative analgesics[2,8]. Irefin et al[9] regarding postoperative pain did not find statistically significant differences between patients with infratentorial craniotomy and patients with supratentorial craniotomy; the median pain score was ≤5. However, we found all 5 patients undergoing decompressive surgery for Chiari malformation had severe postoperative pain. The reason may be due to the extent of the muscles being damaged during surgery. The correlation between preoperative anxiety and postoperative pain has been observed by others as well[16,17]. However, there is no study including craniotomy patients, which is another pointer to why pain in these patients is being generally underestimated. Neurosurgical patients have high levels of anxiety, particularly about surgery but less about anesthesia, as demonstrated by Perks et al[11]. The incidence of preoperative anxiety and high level of anxiety in their patients was 89% and 55% respectively, whereas the

Table 3. Demographic factors associated with moderate to severe pain after craniotomy

Factors	Mean ± SD or number n (%)		Crude OR (95% CI)	*p*-value[1]	Adjusted OR (95% CI)	*p*-value[2]
	NRS <4 n = 70	NRS ≥4 n = 210				
Age (years)				0.011*		
<45	15 (21.4)	83 (39.5)	3.28 (1.43-7.50)		3.165 (1.318-7.600)	0.010*
45-60	39 (55.7)	100 (47.6)	1.52 (0.74-3.12)		1.584 (0.736-3.412)	0.240
>60	16 (22.9)	27 (12.9)	1		1.000	
ASA class I-II	65 (92.9)	197 (93.8)	1.17 (0.40-3.39)	0.778	-	-
ASA class III	5 (7.1)	13 (6.2)	1			
Education				0.196	1.385 (0.707-2.712)	0.343
Below bachelor degree	54 (77.1)	145 (69.0)	1			
Bachelor degree and above	16 (19.8)	65 (31.0)	1.51 (0.81-2.84)			
Preexisting pain				0.214		
No			1			
≤3 months duration	7 (10.0)	40 (19.0)	2.14 (0.89-5.11)		2.027 (0.816-5.038)	0.128
>3 months duration	17 (24.3)	47 (22.5)	1.03 (0.54-1.98)		0.953 (0.473-1.920)	0.893
Perioperative steroids				0.042*	1.751 (0.920-3.333)	0.088
No	17 (24.3)	79 (37.6)	1.88 (1.02-3.47)			
Yes	53 (75.7)	131 (62.4)	1			
APAISa; high level of anxiety (≥10)	8 (11.4)	46 (21.9)	2.30 (1.13-4.67)	0.054	2.129 (0.951-4.768)	0.066
APAISk; need for information (≥3)	33 (47.1)	124 (59.0)	1.62 (0.94-2.79)	0.086	1.008 (0.534-1.900)	0.981
Preoperative expectation of postoperative pain score (NRS 0-10)				0.009*	-	-
Mild (0-3)	26 (37.1)	45 (21.4)				
Moderate to severe (4-10)	44 (62.9)	165 (78.6)				

NRS = numeric rating scale score, ASA = American society of Anesthesiology, APAISa = Amsterdam preoperative anxiety and informative scale anxiety component, APAISk = Amsterdam preoperative anxiety and informative scale knowledge component

p-vale[1] came from univariate analysis, *p*-value[2] came from multiple logistic regression analysis

Table 4. Perioperative factors associated with moderate to severe pain after craniotomy

Factors	Mean ± SD or number n (%)		Crude OR (95% CI)	*p*-value[1]	Adjusted OR (95% CI)	*p*-value[2]
	NRS <4 n = 70	NRS ≥4 n = 210				
Craniotomy site				0.2430	1.417 (0.705-2.850)	0.328
Supratentorial	55 (78.6)	150 (71.4)	1.49 (0.78-2.83)			
Infratentorial	15 (21.4)	60 (28.6)	1			
Local wound infiltration at the end of surgery				0.7960	-	-
No			1			
Yes	14 (33.3)	43 (35.5)	1.10 (0.53-2.32)			
Intraoperative fentanyl (mcg/kg/hour)				0.3260	-	-
≤1	62 (89.8)	178 (84.7)	1			
>1	7 (10.2)	31 (15.2)	1.54 (0.65-3.68)			
Postoperative pain perception compare to preoperative expectation				0.0090*	-	-
Less	51 (72.9)	128 (62.4)				
Same	18 (25.7)	46 (22.4)				
Worse	1 (1.4)	31 (15.1)				
0-24 hour morphine-equivalent dosage consumption (mg)	6.20±3.60	9.30±4.20		<0.0001	-	-
24-48 hour morphine-equivalent dosage consumption (mg)	1.30±1.40	3.10±2.70		0.0100	-	-
Total morphine-equivalent dosage consumption (mg)	6.40±4.00	10.30±5.30		<0.0001	-	-
0-24 hour paracetamol consumption (g)	1.15±0.37	1.27±0.59		0.1900	-	-
24-48 hour paracetamol consumption (g)	1.71±0.86	1.94±0.99		0.2740	-	-

p-value[1] came from univariate analysis, *p*-value[2] came from multiple logistic regression analysis

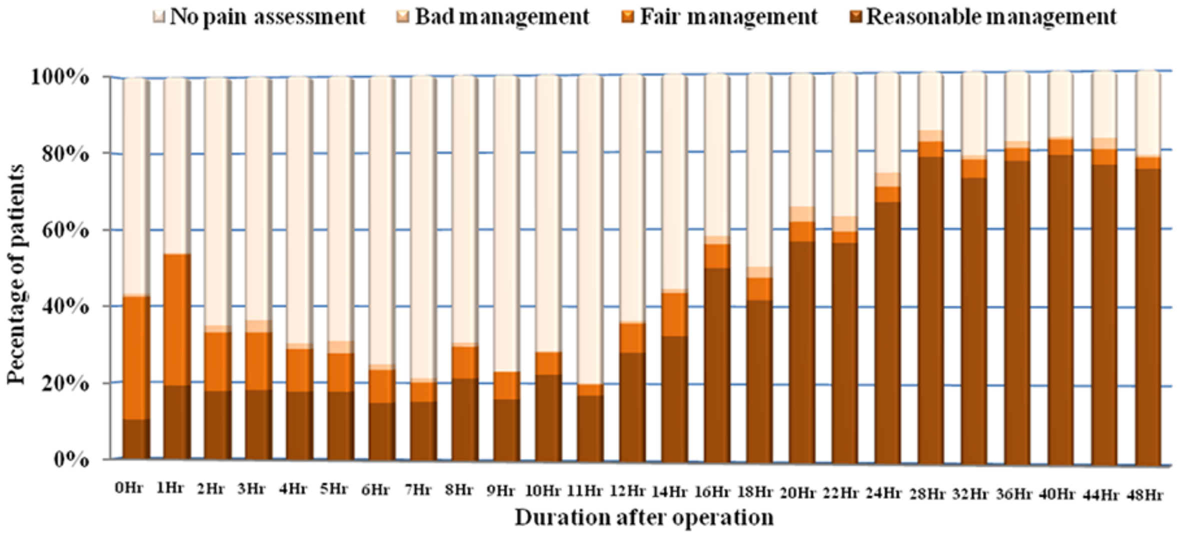

Fig. 2 Pain assessment and quality of pain management during the first 48 postoperative hours.

rate of high level of anxiety in our patients was only 26.4%, and much more likely due to the different cultures studied. Thai patients are normally uncomplaining and consideration to the health personnel. However both studies demonstrated that anxiety was higher in women, indicating in an increased need of information about surgery and anesthesia.

Our study was consistent with other study. Consistent to Hansen et al[15] we found the perioperative steroid usage resulted in better pain relief. They also demonstrated that the mean postoperatively 0-48 hours morphine consumption was 28.80$\pm$23.6 mg, whereas in our study it was only 9.3$\pm$5.3 mg. This might be due to cultural characteristics as described above together with the attitude of the personnel being reluctant giving morphine.

In our study, morphine and acetaminophen were the most common analgesic drugs given during the first and second postoperative day. Codeine is not available in our country. Similar to our observations, Gottschalk et al[2] reported that 80% and 58% of their patients received acetaminophen and intravenous fentanyl, respectively. Codeine has been the main analgesics in neurosurgical units for decades, as it is exemplarily described in the survey of Stoneham et al[4]; 97% of neuro-anaesthetists prescribed intramuscular codeine phosphate or dihydrocodeine for post-operative analgesia. Roberts et al[18] reported that 49%, 22%, and 12% of the neurosurgical units in the United Kingdom prescribed codeine phosphate with acetaminophen, codeine phosphate with acetaminophen and diclofenac and morphine for postoperative analgesia. Also for the United Kingdom, Kotak et al[19] investigated the practice of pain therapy in adult neurosurgical units. They found that 70% of the units used codeine as first line an algesic but only 30% of them used morphine. The reasons to avoid the application of potent opioids included concern about potential side effects, such as respiratory depression and slowing down pupillary reaction. Therefore, pain control after craniotomy is usually inadequate; this is also observed among others as demonstrated in this study.

The ideal analgesic drug for postoperative pain treatment after craniotomy should have no effect on pupil size, respiratory rate, consciousness and platelet function. In our hospital, morphine is the most common intravenous analgesic prescribed on demand during the early postoperative period, whereas 47-70% of the hospitals in Canada and United Kingdom prescribed codeine and only 30-38% prescribed morphine as first line drug[19,20].

Up to date, there are still no 'best analgesics' for postoperative craniotomy pain. Opioids are still the cornerstone of pain treatment. The results of Hansens et al[21] meta-analysis have suggested that scalp infiltration may be a simple alternative providing adequate analgesic effect during the first few postoperative hours. Acetaminophen given routinely around the clock have been demonstrated to be inadequate[22] for postoperative pain relief after craniotomy with a failure rate of 73%[7].

In search of alternative drugs, parecoxib, other COX-2-inhibitors and gabapentin have been

investigated, for instance, by Jones et al[23], Williams et al[24] and Ture et al[25] with conflicting results for parecoxib and concerns about side effects, such as sedation for gabapentin. The frequency of pain assessment in our patients should be improved, especially during the immediate postoperative period. Therefore, we have to improve the education of nurses and therapists taking care of these patients. However, the reason for low pain assessment may be the lack of patient's ability to evaluate pain score. Therefore, using non-verbal pain assessment may be suitable in this situation[26].

Conclusion

In this study, postoperative pain after major craniotomy is more severe as a high number of patients have moderate to severe pain, especially in patients younger than 45 years old. Increasing postoperative care including pain assessment and treatment should be improved in patients after craniotomy. The key to improve pain management is increased concern and provide education on acute postoperative pain management for health personnel.

What is already known on this topic?

Most of the patients undergone major craniotomy suffer moderate to severe pain and they are undertreated. The risk factors for acute postoperative pain are gender, age, anxiety and craniotomy sites.

What this study adds?

Younger patents experience more pain than the elderly do. More attention should be paid to the care of postoperative pain management in patients after major craniotomy.

Acknowledgements

This research is supported by Siriraj Research Development Fund. We would like to thank Prof. Shusee Visalyaputra for her help in reviewing the manuscript, Miss Chulalux Komoltri for her statistical analysis and Miss Nichapat Sooksri for her help in organizing this research.

Potential conflicts of interest

None.

References

1. Dunbar PJ, Visco E, Lam AM. Craniotomy procedures are associated with less analgesic requirements than other surgical procedures. Anesth Analg 1999; 88: 335-40.
2. Gottschalk A, Berkow LC, Stevens RD, Mirski M, Thompson RE, White ED, et al. Prospective evaluation of pain and analgesic use following major elective intracranial surgery. J Neurosurg 2007; 106: 210-6.
3. De Benedittis G, Lorenzetti A, Migliore M, Spagnoli D, Tiberio F, Villani RM. Postoperative pain in neurosurgery: a pilot study in brain surgery. Neurosurgery 1996; 38: 466-9.
4. Stoneham MD, Walters FJ. Post-operative analgesia for craniotomy patients: current attitudes among neuroanaesthetists. Eur J Anaesthesiol 1995; 12: 571-5.
5. Basali A, Mascha EJ, Kalfas I, Schubert A. Relation between perioperative hypertension and intracranial hemorrhage after craniotomy. Anesthesiology 2000; 93: 48-54.
6. de Gray LC, Matta BF. Acute and chronic pain following craniotomy: a review. Anaesthesia 2005; 60: 693-704.
7. Nair S, Rajshekhar V. Evaluation of pain following supratentorial craniotomy. Br J Neurosurg 2011; 25: 100-3.
8. Thibault M, Girard F, Moumdjian R, Chouinard P, Boudreault D, Ruel M. Craniotomy site influences postoperative pain following neurosurgical procedures: a retrospective study. Can J Anaesth 2007; 54: 544-8.
9. Irefin SA, Schubert A, Bloomfield EL, DeBoer GE, Mascha EJ, Ebrahim ZY. The effect of craniotomy location on postoperative pain and nausea. J Anesth 2003; 17: 227-31.
10. Moerman N, van Dam FS, Muller MJ, Oosting H. The Amsterdam Preoperative Anxiety and Information Scale (APAIS). Anesth Analg 1996; 82: 445-51.
11. Perks A, Chakravarti S, Manninen P. Preoperative anxiety in neurosurgical patients. J Neurosurg Anesthesiol 2009; 21: 127-30.
12. Yaksh TL, Wallace MS. Opioid, analgesia, and pain management. In: Brunton LL, Chaner BA, Knollmann BC, editors. Goodmann & Gilman's the pharmacological basis of therapeutics. 12th ed. New York: McGraw Hill; 2011: 481-525.
13. Rasmussen GL, Steckner K, Hogue C, Torri S, Hubbard RC. Intravenous parecoxib sodium foracute pain after orthopedic knee surgery. Am J Orthop (Belle Mead NJ) 2002; 31: 336-43.
14. Mordhorst C, Latz B, Kerz T, Wisser G, Schmidt A,

Schneider A, et al. Prospective assessment of postoperative pain after craniotomy. J Neurosurg Anesthesiol 2010; 22: 202-6.
15. Hansen MS, Brennum J, Moltke FB, Dahl JB. Suboptimal pain treatment after craniotomy. Dan Med J 2013; 60: A4569.
16. Ip HY, Abrishami A, Peng PW, Wong J, Chung F. Predictors of postoperative pain and analgesic consumption: a qualitative systematic review. Anesthesiology 2009; 111: 657-77.
17. Gramke HF, de Rijke JM, van Kleef M, Kessels AG, Peters ML, Sommer M, et al. Predictive factors of postoperative pain after day-case surgery. Clin J Pain 2009; 25: 455-60.
18. Roberts GC. Post-craniotomy analgesia: current practices in British neurosurgical centres—a survey of post-craniotomy analgesic practices. Eur J Anaesthesiol 2005; 22: 328-32.
19. Kotak D, Cheserem B, Solth A. A survey of post-craniotomy analgesia in British neurosurgical centres: time for perceptions and prescribing to change? Br J Neurosurg 2009; 23: 538-42.
20. Hassouneh B, Centofanti JE, Reddy K. Pain management in post-craniotomy patients: a survey of canadian neurosurgeons. Can J Neurol Sci 2011; 38: 456-60.
21. Hansen MS, Brennum J, Moltke FB, Dahl JB. Pain treatment after craniotomy: where is the (procedure-specific) evidence? A qualitative systematic review. Eur J Anaesthesiol 2011; 28: 821-9.
22. Verchere E, Grenier B, Mesli A, Siao D, Sesay M, Maurette P. Postoperative pain management after supratentorial craniotomy. J Neurosurg Anesthesiol 2002; 14: 96-101.
23. Jones SJ, Cormack J, Murphy MA, Scott DA. Parecoxib for analgesia after craniotomy. Br J Anaesth 2009; 102: 76-9.
24. Williams DL, Pemberton E, Leslie K. Effect of intravenous parecoxib on post-craniotomy pain. Br J Anaesth 2011; 107: 398-403.
25. Ture H, Sayin M, Karlikaya G, Bingol CA, Aykac B, Ture U. The analgesic effect of gabapentin as a prophylactic anticonvulsant drug on postcraniotomy pain: a prospective randomized study. Anesth Analg 2009; 109: 1625-31.
26. Topolovec-Vranic J, Canzian S, Innis J, Pollmann-Mudryj MA, McFarlan AW, Baker AJ. Patient satisfaction and documentation of pain assessments and management after implementing the adult nonverbal pain scale. Am J Crit Care 2010; 19: 345-54.

อาการปวดหลังการผ่าตัดใหญ่ของสมองในโรงพยาบาลมหาวิทยาลัย: การศึกษาไปข้างหน้า

ศิริลักษณ์ สุขสมปอง, นพนันท์ ชัยกิตติศิลป์, ทัดรวี รัชฎาวงษ์, เอกวุฒิ จันทร์แก้ว, เบนโน วอน โบแมน

ภูมิหลัง: *อาการปวดหลังการผ่าตัดใหญ่ของสมองได้รับการเชื่อว่ารุนแรงน้อยกว่าอาการปวดจากการผ่าตัดอื่นๆ*

วัตถุประสงค์: *เพื่อศึกษาอุบัติการณ์และปัจจัยเสี่ยงของอาการปวดชนิดปานกลางถึงรุนแรงหลังการผ่าตัดสมอง*

วัสดุและวิธีการ: *เป็นการศึกษาแบบสังเกตชนิดเก็บข้อมูลไปข้างหน้าในหออภิบาลและหอผู้ป่วยของหน่วยศัลยศาสตร์ระบบประสาท ทำการเก็บข้อมูลก่อนผ่าตัดที่ 24 และ 48 ชั่วโมงหลังผ่าตัดผู้ป่วยที่เข้ารับการผ่าตัดสมองระหว่างเดือนพฤษภาคม พ.ศ. 2554 ถึง สิงหาคม พ.ศ. 2555 โดยการสัมภาษณ์ผู้ป่วยก่อนผ่าตัดและที่ 24 และ 48 ชั่วโมงหลังผ่าตัด ทำการบันทึกข้อมูลประชากร คะแนนความกังวลก่อนผ่าตัด ข้อมูลการผ่าตัด และข้อมูลเกี่ยวกับอาการปวดหลังผ่าตัดรวมถึงการรักษา*

ผลการศึกษา: *มีจำนวนผู้ป่วย 280 ราย เข้าร่วมการศึกษา พบอุบัติการณ์ของอาการปวดหลังผ่าตัดสมองชนิดปานกลางถึงรุนแรงร้อยละ 75 ค่าเฉลี่ยของอาการปวดระหว่าง 24 และ 48 ชั่วโมง 5.5±2.7 และ 3.5±2.6 ตามลำดับ จากการวิเคราะห์แบบ univariate analysis พบว่าปัจจัยเสี่ยงต่อการเกิดอาการปวดชนิดปานกลางถึงรุนแรงได้แก่ อายุน้อยกว่า 45 ปี และการได้รับยาสเตอรอยด์ก่อนผ่าตัดจากการวิเคราะห์แบบ multivariate analysis พบว่าปัจจัยเสี่ยงต่อการเกิดอาการปวดชนิดปานกลางถึงรุนแรงได้แก่ อายุน้อยกว่า 45 ปี คะแนนความพึงพอใจต่อการบรรเทาอาการปวดหลังผ่าตัดอยู่ในเกณฑ์ดีในผู้ป่วยกลุ่มที่มีอาการปวดน้อยและอาการปวดชนิดปานกลางถึงรุนแรง (9.49±1.08 และ 8.37±1.76) ในระยะหลังผ่าตัดผู้ป่วยเกือบทุกรายได้รับยากลุ่มโอปิออยด์บริหารทางหลอดเลือดดำและยาอะซีตามิโนเฟนชนิดรับประทานเพื่อบรรเทาอาการปวด ไม่พบภาวะกดหายใจแต่พบอาการคลื่นไส้และอาเจียนร้อยละ 51.7 และอาการคันร้อยละ 23.6*

สรุป: *อุบัติการณ์อาการปวดหลังผ่าตัดสมองพบมากในผู้ป่วยอายุน้อยซึ่งผลนี้แตกต่างจากการศึกษาอื่น การดูแลบรรเทาอาการปวดหลังผ่าตัดสมองควรได้รับการพัฒนาให้ดีขึ้น*
