# Supplementary material for: Postoperative analgesia of scalp nerve block with ropivacaine in pediatric craniotomy patients: a protocol for a prospective, randomized, placebo-controlled, double-blinded trial
Source: Trials. 2020 Jun 26;21:580. doi: 10.1186/s13063-020-04524-7 (PMC7318534; doi:10.1186/s13063-020-04524-7)
Supplement: Supplementary file 1 — Additional file 1. [file 13063_2020_4524_MOESM1_ESM.zip › reference 28R2.pdf]

ResearchGate

See discussions, stats, and author profiles for this publication at: https://www.researchgate.net/publication/13998379

The FLACC: A Behavioral Scale for Scoring Postoperative Pain in Young Children

Article *in* Pediatric nursing · November 1996

Source: PubMed

CITATIONS
965

READS
20,198

4 authors, including:

Sandra Merkel
University of Michigan
42 PUBLICATIONS **3,314** CITATIONS

SEE PROFILE

Terri Voepel-Lewis
University of Michigan
204 PUBLICATIONS **7,640** CITATIONS

SEE PROFILE

Shobha Malviya
University of Michigan
159 PUBLICATIONS **7,007** CITATIONS

SEE PROFILE

Some of the authors of this publication are also working on these related projects:

anesthetic management for children with down syndrome View project

No project View project

All content following this page was uploaded by Terri Voepel-Lewis on 01 April 2014.

The user has requested enhancement of the downloaded file.

Practice Applications of Research

Janice S. Hayes, PhD, RN

The FLACC: A Behavioral Scale for Scoring Postoperative Pain in Young Children

Sandra I. Merkel
Terri Voepel-Lewis
Jay R. Shayevitz
Shobha Malviya

Purpose: To evaluate the reliability and validity of the FLACC Pain Assessment Tool which incorporates five categories of pain behaviors: facial expression; leg movement; activity; cry; and consolability.

Method: Eighty-nine children aged 2 months to 7 years, (3.0±2.0 yrs.) who had undergone a variety of surgical procedures, were observed in the Post Anesthesia Care Unit (PACU). The study consisted of: 1) measuring interrater reliability; 2) testing validity by measuring changes in FLACC scores in response to administration of analgesics; and 3) comparing FLACC scores to other pain ratings.

Findings: The FLACC tool was found to have high interrater reliability. Preliminary evidence of validity was provided by the significant decrease in FLACC scores related to administration of analgesics. Validity was also supported by the correlation with scores assigned by the Objective Pain Scale (OPS) and nurses' global ratings of pain.

Conclusions: The FLACC provides a simple framework for quantifying pain behaviors in children who may not be able to verbalize the presence or severity of pain. Our preliminary data indicates the FLACC pain assessment tool is valid and reliable.

Children frequently lack the verbal and cognitive skills necessary to report physical discomfort and pain intensity. Assessment and effective management of pain in this population, therefore, depends upon the observation and expertise of the care provider. Difficulty in pain assessment frequently leads to undertreatment of pain in children (Schechter, 1989). Specific distress behaviors: cry/communication, facial expression, and body/motor movement, have been used to define and describe pain in young children (Davis, 1990; Jay, Ozolins, Elliot & Caldwell, 1984; Johnston, 1989; Katz, Kellerman, & Seigel, 1980; LeBaron & Zeltzer, 1984; Mills, 1989; Taylor, 1983). To facilitate the objective measurement of pain, clinicians and researchers have incorporated these behaviors into scales. The Children's Hospital of Eastern Ontario Pain Scale (CHEOPS) (McGrath et al., 1985), the Objective Pain Scale (OPS) (Norden et al., 1991; Broadman, Rice & Hannallah, 1988), and the Toddler-Preschool Postoperative Pain Scale (TPPPS) (Tarbell, Cohen, & March, 1992) are three such tools that have been tested and reported in the literature. Difficulty in distinguishing pain from other distress behaviors led one group of investigators to include categories for sleep, suck, and consolablility in their infant scoring system (Barrier, Attia, Mayer, Amiel-Tison, & Schnider, 1989).

The CHEOPS, OPS, and Barrier/Attia infant scales provide a systematic structure for assessment and documentation of pain in young children. However, the use of these tools in a busy clinical setting is limited because they are lengthy and/or cumbersome to score. The FLACC assessment tool (see Table 1) was developed with input from clinicians in order to provide a simple, consistent method for physicians and nurses to identify, document, and evaluate pain. The FLACC tool incorporates five categories of behavior previously used in other scales. The acronym FLACC (face, legs, activity, cry, and consolability) was devised to facilitate recall of the categories included in the tool. Each category is scored on a 0-2 scale which results in a total score between 0 and 10, a range often found in other clinical assessment tools.

The specific aims of the present study were to determine interrater reliability, to test the validity of the FLACC tool by

Sandra I. Merkel, MS, RN, is a Clinical Nurse Specialist, Department of Anesthesiology, University of Michigan Medical School and Health Systems, Ann Arbor, MI.

Terri Voepel-Lewis, MS, RN, is a Clinical Nurse Specialist, Department of Anesthesiology, University of Michigan Medical School and Health Systems, Ann Arbor, MI.

Jay R. Shayevitz, MD, is Director, Pediatric Perioperative and Critical Care, Providence Hospital and Medical Center, Southfield, MI.

Shobha Malviya, MD, is Assistant Professor, Department of Anesthesiology, University of Michigan Medical School and Health Systems, Ann Arbor, MI.

Note: This content was presented in part at the International Symposium on Pediatric Pain in Philadelphia, PA, 1994; and at the Annual meeting of the American Society of Anesthesiologists, San Francisco, CA, 1994.

The *Practice Applications of Research* section presents reports of research that are clinically focused and discuss the nursing application of the findings. If you are interested in author guidelines and/or assistance, contact Janice S. Hayes, PhD, RN; Section Editor; Pediatric Nursing; East Holly Avenue Box 56; Pitman NJ 08071-0056; (609)256-2300.

Table 1. FLACC Scale

Categories	Scoring		
	0	1	2
Face	No particular expression or smile	Occasional grimace or frown, withdrawn, disinterested	Frequent to constant quivering chin, clenched jaw
Legs	Normal position or relaxed	Uneasy, restless, tense	Kicking, or legs drawn up
Activity	Lying quietly, normal position, moves easily	Squirming, shifting back and forth, tense	Arched, rigid or jerking
Cry	No cry (awake or asleep)	Moans or whimpers; occasional complaint	Crying steadily, screams or sobs, frequent complaints
Consolability	Content, relaxed	Reassured by occasional touching, hugging or being talked to, distractable	Difficult to console or comfort
Each of the five categories (F) Face; (L) Legs; (A) Activity; (C) Cry; (C) Consolability is scored from 0-2, which results in a total score between zero and ten.			

Table 2. Demographic Data

Study Group	Part 1<br>Interrater Reliability	Part 2<br>Analgesic Effect	Part 3<br>Comparison of FLACC to OPS
Subjects n = 89<br>Gender<br><br>Age Group	30<br>m = 14<br>f = 16<br><1 year = 7<br>1-3 years = 19<br>4-7 years = 4	29<br>m = 17<br>f = 12<br><1 year = 4<br>1-3 years = 13<br>4-7 years = 12	30<br>m = 16<br>f = 12<br><1year = 5<br>1-3 years = 14<br>4-7 years = 9<br>*missing data = 2
Surgical Procedure	ENT = 11<br>GU/hernia = 5<br>Opthy = 6<br>Abdominal = 1<br>Lower Extremity = 0<br>Soft Tissue = 2<br>Facial/dental = 3<br>Procedure = 2	ENT = 5<br>GU/hernia = 5<br>Opthy = 3<br>Abdominal = 3<br>Lower Extremity = 5<br>Soft Tissue = 4<br>Facial/dental = 0<br>Procedure = 4	ENT = 3<br>GU/hernia = 6<br>Opthy = 6<br>Abdominal = 1<br>Lower Extremity = 2<br>Soft Tissue = 4<br>Facial/dental = 3<br>Procedure = 2<br>Thoracic = 1<br>Neuro = 1<br>* missing data = 1

measuring changes in FLACC scores in response to administration of analgesics, and to compare the FLACC to other pain assessment tools.

Methods

The study was approved by the Institutional Review Board and informed consent was obtained from parents. Eighty nine children aged 2 months to 7 years,(3.0±2.0 years) who had undergone a variety of elective surgical procedures, were studied in the Post Anesthesia Care Unit (PACU) after completion of surgery (see Table 2). Children with known developmental delay were excluded. After the children were awake or easily arousable from sleep, observations were made by two of the investigators and PACU nurses experienced in the use of behavioral pain scales. Training in the use of the FLACC included discussion of the

five categories, clarification of the defining characteristics for specific behaviors, and scoring.

The study consisted of three parts. The first part involved thirty children and tested interrater reliability of the FLACC tool. After each child was awake and easily aroused, three observations at 5-minute intervals were completed, which provided a total of 90 observations. FLACC scores were simultaneously but independently assigned by two of the investigators for each observation. The investigators did not discuss or share observations or ratings of behaviors. At the last observation for each patient, the PACU nurse caring for the child assigned a global pain rating using a 0-10 number scale (0 = no pain; 10 = the worst pain). The PACU nurses were blinded to the FLACC scores assigned by the investigators.

Validity of the FLACC was tested in the second part of the study. Another group of 29 children was observed before and after opioid analgesic administration. A decision to administer analgesics was made by the nurse and the anesthesiologist caring for the child based on clinical observation and routine pain assessment. The PACU nurses assigned FLACC scores immediately prior to the administration of analgesics and again at 10, 30, and 60 minute intervals after intravenous opiates, or at 30 and 60 minutes after oral acetaminophen or oral codeine. Neither subjects nor observers were blinded to analgesic administration.

Validity was further tested by a comparison of FLACC scores to OPS scores in a third group of thirty children. FLACC scores were assigned by one of the investigators while OPS scores were simultaneously assigned by the other investigator (each observer assigned 15 OPS scores and 15 FLACC scores). Each observer was blinded to the ratings of the other.

Analysis. Two-way cross tabulations and kappa statistics were used to determine interrater reliability. The kappa statistic is a measure of agreement that allows for observer variability for categorical data and corrects for chance levels of agreement. For most purposes, kappa values greater than 0.75 represent agreement not achievable by chance alone and values below 0.40 may be interpreted as poor agreement beyond chance (Fleiss, 1981). Acceptable interrater reliability is indicated by values over 0.41. Correlation coefficients were used to compare the pain scores obtained using the FLACC and OPS assessment tools. Analysis of variance for repeated measures was used to compare FLACC scores before and after analgesic administration. Results of $p<0.05$ were accepted as statistically significant.

Results

Interrater reliability. There was a high correlation between the two observers' FLACC scores ($r[87]=0.94$; $p<0.001$), demonstrating good interrater reliability. In addition, interrater reliability was acceptable as indicated by kappa values of above 0.50 for each category (see Table 3).

Validity. Preanalgesia FLACC scores (7.0 ± 2.9) were significantly higher than the postanalgesia scores at 10 minutes (1.7±2.2), 30 minutes (1.0±1.9), and 60 minutes (0.2±0.5) ($p<0$ .001 for each time interval) (see Figure 1). Analysis of variance did not find significant differences among the 10, 30 and 60 minute scores. There was a positive correlation between total FLACC scores and the PACU nurses' global ratings of pain ($r[47] = 0.41$; $p<0.005$). OPS and FLACC scores had a significant positive correlation indicating that both tools were capturing similar behaviors ($r=.80$; $p<0.001$) (see Figure 2).

Discussion

Reliability and validity of pain assessment tools are

Figure 1. Changes in FLACC scores in response to analgesia. Scores were assigned prior to the administration of analgesia (PRE) and again at 10, 30, and 60 minute intervals after the analgesia (IV morphine or p.o. codeine/acetaminophen) was given.

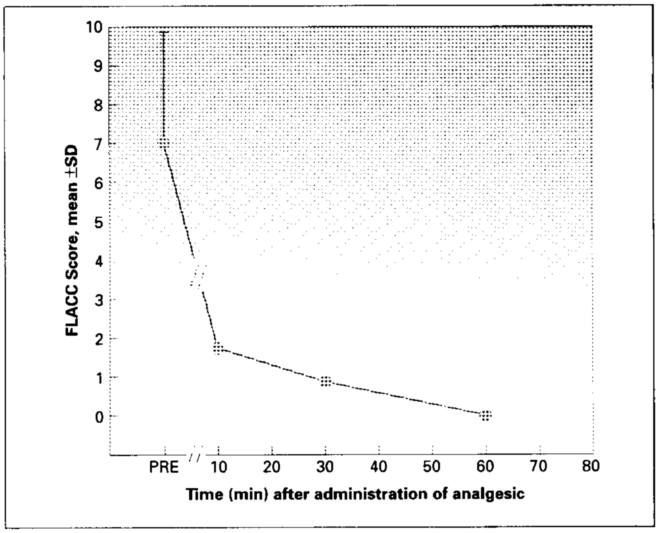

Table 3. Interrater Reliability of the FLACC tool

FLACC Category	(%) Agreement Between Observers	Kappa statistic[a]
Face	69%	0.52
Legs	87%	0.67
Activity	89%	0.72
Cry	91%	0.82
Consolability	78%	0.66

[a]Values over 0.41 are considered indicative of acceptable levels of interrater reliability.

Figure 2. Comparison of FLACC and OPS scores. Solid line represents linear regression relationship between FLACC scores and OPS score

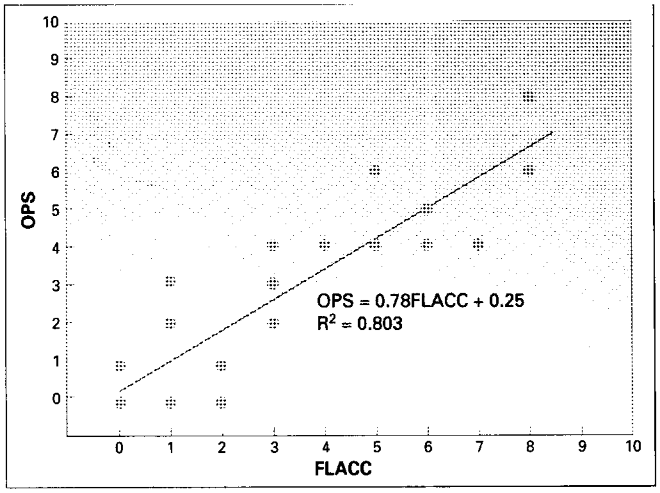

Table 4. Categories of Behavior in Pain Assessment Tools

FLACC	CHEOPS	OPS	TPPPS	Büttner/Finke
Face	Facial expression		Facial pain expression	Facial expression
Legs	Leg movement	Movement		Leg position
Activity	Torso movement	Agitation	Bodily pain expression	Position of torso
				Motoric restlessness
Cry	Cry	Cry	Vocal pain expression	Cry
Consolability	Touching of the wound	Blood pressure		Consolability
	Verbal report of pain	Verbal complaint and body language		

Behavioral categories included in pediatric pain assessment tools, (Children's Hospital of Eastern Ontario Pain Scale [CHEOPS]), Objective Pain Scale (OPS), Toddler-Preschool Postoperative Pain Scale (TPPPS), and the Büttner and Finke study, are similar to the categories in FLACC.

important criteria when selecting tools for use in clinical practice and for research purposes. Pain assessment tools that were previously reported as being reliable and valid were not being used in clinical practice at our institution. Nurse clinicians from six pediatric inpatient units had implemented the use of CHEOPS, OPS and the Attia infant scale for routine pain assessment. The length of the tool and confusing scoring systems were reported as being the major reasons for not using these tools. These three tools were analyzed for similarities in categories, content validity, ease of use, and scoring method. Five categories were selected and a short and simple 0-10 scale was devised so that the assessment tool could be easily incorporated into practice and would promote consistent documentation. This tool was then piloted on the infant unit, PACU, and thoracic surgery unit and final revisions were made in the descriptors used in the categories.

The content validity of the categories in the FLACC assessment tool was established by selecting behaviors described and validated in tools such as CHEOPS, OPS, and other observational studies in the literature (see Table 4). The CHEOPS is used for children between 1 and 7 years of age and requires scoring of six categories: cry, facial expression, verbal expression, torso movement, touching of the wound, and leg movement (McGrath et al., 1985). The OPS tool is similar to the CHEOPS but incorporates only four categories and requires documentation of a percentage change in blood pressure from baseline. Validity of OPS as measure of severe pain has been established in children aged 13 to 18 years by a high correlation of OPS scores with self-report using a linear analogue scale (Broadman et al., 1988). However, this study also found that OPS scores were less valid in the presence of mild or moderate pain (Broadman et al., 1988). The TPPPS is a tool that requires scoring in three general categories: vocal pain expression, facial pain expression, and bodily expression. A recent evaluation of the TPPPS suggests that the scale has good reliability, evidence of validity, sensitivity to analgesic regimens, and convergence between ratings of pain from nurses and parents. The FLACC includes the categories of crying, facial expression, position of trunk, leg position, motoric restlessness, and consolability, which were reported by Büttner and Finke (1991) to be reliably associated with pain.

Results from this three part study provide preliminary evidence of reliability and validity of the FLACC pain assessment tool. In addition to the high correlation between scores of the two raters, interrater agreement for the FLACC is similar to agreement reported for the CHEOPS (Range = 61%-100%) (McGrath et al., 1985). Kappa values of FLACC observations (see Table 3) indicate acceptable agreement and are very similar to those reported for TPPPS (range = 0.53-0.78) (Tarbell et al., 1992). The reproducibility and consistency of scores demonstrated in our study provides confidence about data collected using the FLACC tool.

Construct validity of the FLACC tool as a measure of pain was supported by the significant reductions in scores after analgesic administration. Similar reductions in pain scores after analgesics were found with CHEOPS (McGrath et al., 1985), TPPPS (Tarbell et al., 1992), and the categories defined by Büttner and Finke (1991). Validity was further established by the high correlation of FLACC scores with the OPS scores and nurse's global pain ratings. The OPS was chosen for comparison in this study because of its reported interrater reliability, validity, similarities in categories, and its correlation with self-report of pain in a study of 5 to 13-year-old ICU patients (West et al., 1994).

The methodological limitations of this study require that the results be interpreted as preliminary evidence of validity of the FLACC tool. Sedative properties of opioid analgesics and potential residual sedation from general anesthetics may have complicated the interpretation of behavioral observations, and may have contributed to lower pain

scores. Observers were not blinded to the administration of postoperative analgesia nor were the analgesic regimens controlled. Further more, generalization of our findings to other settings is limited since testing occurred only in the PACU setting. Validity of the FLACC tool could be confirmed by studies that control for analgesic regimens or eliminate the effect of sedation by using non-opioid analgesics. The results of this study could be enhanced by testing the FLACC scale with a population undergoing similar surgical procedures and in an environment where residual anesthetics and sedation are less of an issue. The study design would be further strengthened if pain assessments were completed by observers blinded to the type of analgesic given. Additionally, evaluation of the FLACC in the pediatric intensive care unit would be beneficial since children in this setting are frequently unable to report their pain. The examination of verbal reports of pain in relationship to FLACC scores in older children would also aid in the validation of this behavioral tool. Before generalized application of the FLAAC can occur, continued evaluation of the FLACC tool and its applicability in diverse clinical situations and patient populations is recommended. A comparison of FLACC scores to self report pain scores is currently underway at our center.

Behavioral measures have been used for the assessment of pain both in the clinical as well as in the research setting. Recent trials that evaluated the efficacy of local anesthetics in decreasing pain associated with vaccinations utilized behavioral tools to assess pain (Taddio, Nulman, Koren, Stevens, & Koren, 1995). While there is reported disparity between observed pain behaviors and self-report of pain (Beyer, McGrath, & Berde, 1991), behavioral cues remain the primary indicators of pain in children who are unable to report pain or to follow instructions in the use of assessment tools. Tyler, Tu, Douthit, & Chapman (1993)reported a good correlation among CHEOPS, FACES scale, OUCHER, and an observer scale, suggesting that the tools were measuring similar phenomena. The present data using the FLACC tool provide additional support for the use of behavioral observations in quantifying pain in young children. It must be recognized, however, that behavioral distress unrelated to pain may also be captured by observation tools (McGrath, deVaber, & Hearn, 1985). Whenever feasible, behavioral measurement of pain should be used in conjunction with children's self-report. When self-report is not possible, interpretation of pain behaviors and decision making regarding treatment of pain requires careful consideration of the context of behaviors. West et al.(1994) have documented the benefits of self-report of pain in 5-13 year old oncology patients in an ICU setting, but recommend a continued effort to develop observational pain scales for those children unable to report pain. The FLACC assessment tool was developed to meet this need for observational tools required for pain assessment in nonverbal children.

This study of the FLACC assessment tool contributes data to the overall evaluation of pain measurement in children who cannot report pain. Our results confirm interrater reliability and suggest preliminary validation of the FLACC assessment tool. The FLACC provides a simple framework for quantifying behavioral observation both for research as well as for clinical purposes. We have found the FLACC easy to use and incorporate into documentation which may provide an advantage over other behavioral pain assessment tools. Utilization of the FLACC to assist with the assessment and documentation of pain may facilitate treatment and improve patient outcomes.

References

Barrier, G., Attia, J., Mayer, M.N., Amiel-Tison, C., & Schnider, S.M.(1989). Measurement of postoperative pain and narcotic administration in infants using a new clinical scoring system. *Intensive Care Medicine*, Suppl 1, S37-39.

Beyer, J.E., McGrath, P.J., & Berde, C.B. (1991). Discordance between self-report and behavioral pain measures in children age 3-7 years after surgery. *Journal of Pain and Symptom Management*, *5*, 350-355.

Broadman, L.M., Rice, L.J., & Hannallah, G. (1988). Comparison of a physiologic and a visual analogue pain scale in children. *Canadian Journal of Anaesthesiology, 35*, S137.

Büttner, R., & Finke, W. (1991). Validity and reliability of a scoring system for assessment of postoperative pain in small children. *Journal of Pain and Symptom Management*, *6*, 171.

Davis, K.L. (1990). Postoperative pain in toddlers: Nurses' assessment and intervention. In D.C. Tyler & E.J. Krane (Eds.), *Advances in Pain Research Therapy* (pp. 53-61). New York: Raven Press.

Fleiss, J.L. (1981). *Statistical methods for rates and proportions.* New York: John Wiley and Sons.

Jay, S.M., Ozolins, M., Elliott, C.H., & Caldwell, S. (1984). Assessment of children's distress during painful medical procedures. *Health Psychology, 2*, 133-147.

Johnston, C.C. (1989). Pain assessment and management in infants. *Pediatrician, 16*, 16-23.

Katz, E.R., Kellerman, J., & Seigel, S.E. (1980). Behavioral distress in children with cancer undergoing medical procedures: Developmental considerations. *Journal of Consult Clinical Psychology, 48*, 356-365.

LeBaron, S., & Zeltzer, L. (1984). Assessment of acute pain and anxiety in children and adolescents by self-reports, observer reports and a behavior checklist. *Journal of Consult Clinical Psychology, 52*, 729-738.

McGrath, P.A., deVaber, L.L., & Hearn, M.T. (1985). Multidimensional pain assessment in children. *Advances in Pain Research and Therapy, 9*, 387-393.

McGrath, P.J., Johnson, G., Goodman, J.T., Schillinger, J., Dunn, J., & Chapman, J. (1985). CHEOPS: A behavioral scale for rating postoperative pain in children. In H.L. Fields (Ed.), *Advances in Pain Research and Therapy* (pp. 395-402). New York: Raven Press.

Mills, N. (1989). Pain behavior in infants and toddlers. *Journal of Pain and Symptom Management*, *4*, 184-190.

Norden, J., Hannallah, R., Getson, P., O'Donnell, R., Kelliher, G., & Walker, N. (1991). Reliability of an objective pain scale in children. *Journal of Pain and Symptom Management*, *6*, 196.

Schechter, N.L. (1989). The undertreatment of pain in children: An overview. *Pediatric Clinics of North America, 36*, 781-794.

Taddio, A., Nulman, I., Koren, B.S., Stevens, B., & Koren, G. (1995). A revised measure of acute pain in *infants. Journal of Pain and Symptom Management*, *10*, 456-463.

Tarbell, S.E., Cohen I.T., & March, J.L. (1992). The Toddler-Preschool Postoperative Pain Scale: An observational scale for measuring postoperative pain in children aged 1-5. Preliminary report. *Pain, 50*, 273-280.

Taylor, P.L. (1983) Post-operative pain in toddler and pre-school age children. *Maternal Child Nursing, 12*, 5-50.

Tyler, D.C., Tu, A., Douthit, J., & Chapman, C.R. (1993). Toward validation of pain measurement tools for children: A pilot study. *Pain, 52*, 301-309.

West, N., Oakes, L., Hinds, P.S., Sanders, L., Holden, R., Williams, S., Fairclough, D., & Bozeman, P. (1994). Measuring pain in pediatric oncology ICU patients. *Journal of Pediatric Oncology Nursing, 11*, 64-68.

View publication stats
