# Supplementary material for: Postoperative analgesia of scalp nerve block with ropivacaine in pediatric craniotomy patients: a protocol for a prospective, randomized, placebo-controlled, double-blinded trial
Source: Trials. 2020 Jun 26;21:580. doi: 10.1186/s13063-020-04524-7 (PMC7318534; doi:10.1186/s13063-020-04524-7)
Supplement: Supplementary file 1 — Additional file 1. [file 13063_2020_4524_MOESM1_ESM.zip › reference 3R2.pdf]

# Craniotomy Procedures Are Associated with Less Analgesic Requirements than Other Surgical Procedures

Peter J. Dunbar, MB, ChB*, Elizabeth Visco, CRNA*, and Arthur M. Lam, MD, FRCPC*†

Departments of *Anesthesiology and †Neurological Surgery, Harborview Medical Center, University of Washington School of Medicine, Seattle, Washington

The conventional wisdom that neurosurgical patients experience minimal postoperative pain and require little analgesia has been challenged. To address this, we reviewed our anesthesia and postanesthesia care unit (PACU) records for 1995 and compared pain management in patients undergoing major intracranial and selected extracranial procedures. We recorded patient weight, operative time, time in the PACU, intraoperative and postoperative opioid use, PACU pain scores, and level of consciousness in patients who had undergone open fixation of mandible or maxilla (Group E), clipping of aneurysms or excision of tumors (Group I), or lumbar laminectomy (Group L). Group I ($n$ = 78) patients received less fentanyl in the operating room (0.016 $\mu g \cdot kg^{-1} \cdot min^{-1}$ versus 0.023 $\mu g \cdot kg^{-1} \cdot min^{-1}$ for Group E [$n$ = 134] and 0.023 $\mu g \cdot kg^{-1} \cdot min^{-1}$ for Group L [$n$ = 21]; $P < 0.05$), received less morphine in the PACU (0.0004 vs 0.0013 vs 0.0015 $mg \cdot kg^{-1} \cdot min^{-1}$; $P < 0.005$), reported lower pain scores (0.76 vs 2.5 vs 2.4; $P < 0.05$), and spent less time in the PACU (89.5 vs 109 vs 105 min; $P < 0.05$) than Group E or L patients. Our results were similar when only patients with Glasgow Coma Scale scores ≥14 were used in a subset analysis. We conclude that patients suffer less pain and use fewer opioids in the PACU after intracranial surgery than after facial reconstruction or lumbar laminectomy. Our results confirm that the average craniotomy patient has less postoperative pain than patients who undergo other surgical procedures, although patients who undergo frontal craniotomy may require more aggressive pain management. **Implications:** This study compares the pain report and analgesic use in patients after intracranial versus extracranial surgery. The results confirm the commonly held but recently challenged belief that neurosurgery patients suffer less pain postoperatively than other patients. In this study, we found that most patients report minimal pain after intracranial surgery but that a small subset of patients, many of whom have undergone frontal craniotomies, require aggressive treatment of postoperative pain.

(Anesth Analg 1999;88:335–40)

The conventional wisdom that most neurosurgical patients have reduced analgesic requirements was recently challenged (1–3). Stoneham and Walters (2) surveyed 183 British neuroanesthetists, and more than half of the 103 valid respondents believed that postoperative neurosurgical pain was undertreated. De Benedittis et al. (1) reported that 60% of their craniotomy patients suffered pain; in two thirds of their patients, the pain was either moderate or severe. They also noted that pain was more common in women and in patients who had undergone subfrontal and subtemporal surgical approaches. They observed that their results are in direct contrast with the historically accepted 5%–15% incidence of postoperative pain after neurosurgical intracranial procedures (4).

It was our clinical impression that the neurosurgical patients admitted to the postanesthesia care unit (PACU) do not complain of pain as much as other patients. This observation seemed to be independent of the patient's mental status.

Based on our experience, we hypothesized that patients undergoing brain surgery have significantly less postoperative pain than patients undergoing other surgical procedures. Although recent articles have reported significant postoperative pain (1,2), they did not compare brain surgical procedures with other surgical procedures. Without a control group, it is difficult to draw valid conclusions. To test this hypothesis, we retrospectively reviewed the charts of all patients in our institution who underwent major intracranial, facial bone, and lumber laminectomy surgery during 1995.

Accepted for publication November 11, 1998.

Address correspondence to Peter J. Dunbar, MB, ChB, Department of Anesthesiology, Box 359724, Harborview Medical Center, 325 Ninth Ave., Seattle, WA 98104-2499. Address e-mail to pjdunbar@u.washington.edu.

Address reprint requests to Arthur M. Lam, MD, Box 359724, Harborview Medical Center, 325 Ninth Ave., Seattle, WA 98104-2499.

©1999 by the International Anesthesia Research Society
0003-2999/99/$5.00

## Methods

After approval from our human subjects review committee, we retrospectively reviewed the anesthesia and PACU records of all patients who had undergone intracranial procedures; open fixation of mandible, maxilla, or zygomatic arch; and lumbar laminectomies during 1995. Charts were selected for review according to the Current Procedural Terminology code as recorded by the billing records. Patients admitted directly to the intensive care unit or transferred to radiology were excluded from analysis, as were those who had received regional nerve blocks or neuraxial opioid analgesia. We divided the patients into three groups according to the nature of the procedure. Group E included those who had undergone open reduction and fixation of mandible, maxilla and zygoma; Group I included those who had undergone intracranial surgery for aneurysm, tumor, or arteriovenous malformation; and Group L included those who had undergone decompressive lumbar laminectomy.

Although our primary interest was in the patients' postoperative analgesic needs, we also examined intraoperative opioid administration, as this could clearly influence the amount of postoperative pain. PACU nurses routinely recorded pain scores (0–10 on a verbal analog score) in addition to mentation, eye-opening, best verbal response, and best motor response from upper and lower extremities on admission to the PACU and every 15 min thereafter. We used these observations to calculate the Glasgow Coma Scale (GCS) score. We analyzed the score recorded 15 min after admission to the PACU because it was recorded after a period of stabilization.

The PACU nurses routinely assessed objective and subjective pain scores. Alert and oriented patients rated their pain on a 0–10 scale and were asked whether they wanted analgesia. Nurses subjectively assessed, through observation of acute pain behaviors, patients who were unable to describe their pain due to level of consciousness or other communication impairment.

Our attending anesthesiologists most often gave fentanyl as part of their anesthetic in the operating room (OR) and prescribed IV morphine for analgesia in the PACU. For comparative analysis, all intraoperative opioids other than fentanyl were converted to fentanyl equivalents: 1 mg of morphine = 25 $\mu$g of fentanyl, 1 $\mu$g of sufentanil = 5 $\mu$g of fentanyl, and 10 $\mu$g of alfentanil = 1 $\mu$g of fentanyl (5). No effort was made to correct for either time of administration or other pharmacokinetic variables, but, as a rule, morphine was given toward the end of the operation, whereas sufentanil and fentanyl were administered during the first 2–3 h of surgery. In the PACU, the nurses administered opioid (equivalent to a 1- to 4-mg morphine bolus) as often as necessary to all patients whose pain scores were unacceptable to the patient or on patient request. There was no arbitrary upper limit to morphine use in the PACU. To allow comparisons among groups, the postoperative opioid was expressed as morphine equivalents: fentanyl = 40x morphine, meperidine = 0.25x morphine, and ketorolac = 0.2x morphine (5). We then compared the use of opioid, both in total amount and use per unit of time, among the three groups. To adjust for patient weight, the rate of opioid use is expressed in milligrams per kilogram per minute. To eliminate potential systematic bias introduced by differences in the level of consciousness, we reanalyzed a subset of data from patients in Groups I and E who had a GCS score of 14 or 15 in the PACU after the global analysis.

We used JMP (SAS Institute Inc., Cary, NC) software for statistical analysis. The "means diamonds" plots represents the sample size by width of the diamond, and "height" values describe the 95% confidence interval. For statistical testing, Student's *t*-tests, analysis of variance, and Wilcoxon ranked sum test with Bonferroni correction were used as appropriate. Significance was set at $P < 0.05$.

## Results

We retrieved 300 patient records: 153 in Group E, 120 in Group I, and 27 in Group L. After excluding patients who were not admitted to the PACU and one patient who received epidural morphine, we had 234 records for analysis. Table 1 shows group means for weight, operative time and PACU time, and the number of records with documented pain and GCS scores for each group.

Most patients received only fentanyl intraoperatively. In Group I, 4 patients received sufentanil, 1 received alfentanil, and 1 received both fentanyl and morphine. In Group E, 16 patients received both fentanyl and morphine, 4 received morphine alone, and 1 received both fentanyl and sufentanil. In Group L, 2 patients received sufentanil. In the postoperative period, most patients received morphine. In addition, 29 patients in Group E received meperidine, 23 patients received fentanyl, and 21 patients received ketorolac; in Group I, 10 patients received meperidine and 4 patients received fentanyl; in Group L, 3 patients received meperidine, 3 patients received fentanyl, and 1 patient received ketorolac.

The duration of intracranial neurosurgical procedures was significantly longer compared with that of other groups ($P < 0.0001$), but the PACU times were significantly shorter. Total intraoperative fentanyl use in absolute doses was similar in all groups (E = 343 $\mu$g, I = 514 $\mu$g, L = 351 $\mu$g). Because of the

**Table 1.** Demographics, Pain Scores, and Opioid Use

| | Group E (extracranial) | Group I (intracranial) | Group L (lumbar) |
|---|---|---|---|
| Charts reviewed | 153 | 120 | 27 |
| Patients enrolled | 134 | 78 | 21 |
| Weight (kg) | 73 ± 1 | 74 ± 2 | 80 ± 3 |
| OR time (min) | 230 ± 14 | 487 ± 17 | 222 ± 14 |
| OR fentanyl ($\mu g \cdot kg^{-1} \cdot min^{-1}$) | 0.023 ± 0.0001 | 0.016 ± 0.001* | 0.023 ± 0.003 |
| PACU time (min) | 110 ± 5 | 90 ± 3 | 105 ± 4 |
| PACU morphine ($mg \cdot kg^{-1} \cdot min^{-1}$) | 0.0013 ± 0.0001 | 0.0004 ± 0.0001* | 0.0015 ± 0.0001 |
| PACU pain scores recorded[a] | 124 | 77 | 19 |
| PACU GCS score recorded[a] | 98 | 69 | 18 |
| PACU pain scores | 2.5 ± 0.2 | 0.76 ± 0.3 | 2.4 ± 0.6 |

All values are mean ± SE.
OR = operating room, PACU = postanesthesia care unit, GCS = Glasgow Coma Scale.
* $P < 0.05$.
[a] Not all records reviewed were properly completed.

different durations in OR time, all subsequent analyses were adjusted for weight as well as for time (E = 0.023 $\mu g \cdot kg^{-1} \cdot min^{-1}$, I = 0.016 $\mu g \cdot kg^{-1} \cdot min^{-1}$, L = 0.023 $\mu g \cdot kg^{-1} \cdot min^{-1}$). The same methodology was used to compare PACU morphine use (Table 1).

Group I patients reported less pain and received fewer opioids in the PACU ($P < 0.0005$) than either Group E or L patients. There was no difference in either pain scores or opioid use between Groups E and L in the PACU (Table 1). The pain scores were normally distributed in Groups E and L, but they were skewed in Group I (Figure 1). No patients were receiving $\alpha_2$-agonists, but all 31 aneurysm patients were receiving nimodipine and dilantin prophylaxis. Because the results from the aneurysm patients were similar to those from other Group I patients, they were analyzed as a group.

We analyzed the GCS scores as a measure of quantifying the level of consciousness. Comparison of the histogram plots of the GCS score distribution for the intracranial and extracranial groups showed that the mean scores were disproportionately affected by outliers. The intracranial group had more individuals with compromised GCS scores than the other groups (Figure 2). Although it is reasonable to assume that the GCS score must have been ≥14 in patients in whom it was not documented, we restricted the histogram plot to the patients with documented scores.

To avoid underestimating pain perception and analgesic requirements due to a depressed level of consciousness, we reanalyzed a subset of the patients ($n$ = 31 in Group I, $n$ = 84 in Group E) who had recorded GCS scores of 14 or 15 in the PACU. The subset analysis did not differ from analysis of the complete data set; i.e., Group I patients had lower pain scores and required fewer opioids. Because of the smaller number and non-Gaussian distribution, nonparametric analysis was used for this subset data analysis (Figure 3). The subset analysis also revealed that

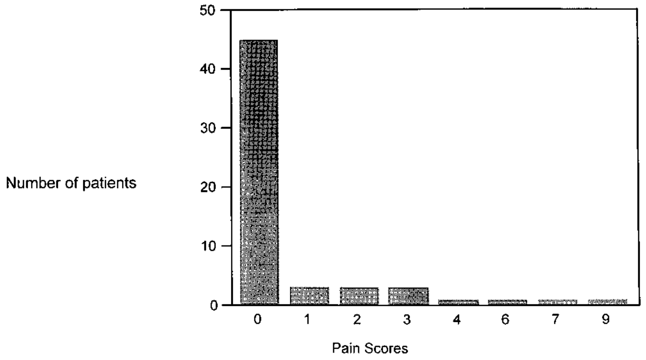


**Figure 1.** Most intracranial surgery patients reported no postoperative pain to postanesthesia care unit nurses. A small number did report severe to moderate pain.

allpatients in Group I with GCS scores >14 who reported pain scores >4 or used >10 mg of morphine in the PACU had undergone frontal craniotomies (Table 2).

## Discussion

Our results demonstrate that neurosurgical patients who undergo intracranial procedures report less pain and require less opioid analgesia than patients undergoing major facial surgery or lumbar laminectomy. Furthermore, neurosurgical patients who are awake and, therefore, presumably capable of full pain perception and expression in the recovery room, suffer less pain and require fewer opioids than a comparable group of patients who undergo surgery on facial bones or lumbar laminectomies. These findings are consistent with many institutional beliefs and practices but are at variance with the results of De Benedittis et al. (1).

De Benedittis et al. (1) studied 37 patients undergoing various intracranial surgical procedures and reported that 40% of the patients experienced moderate

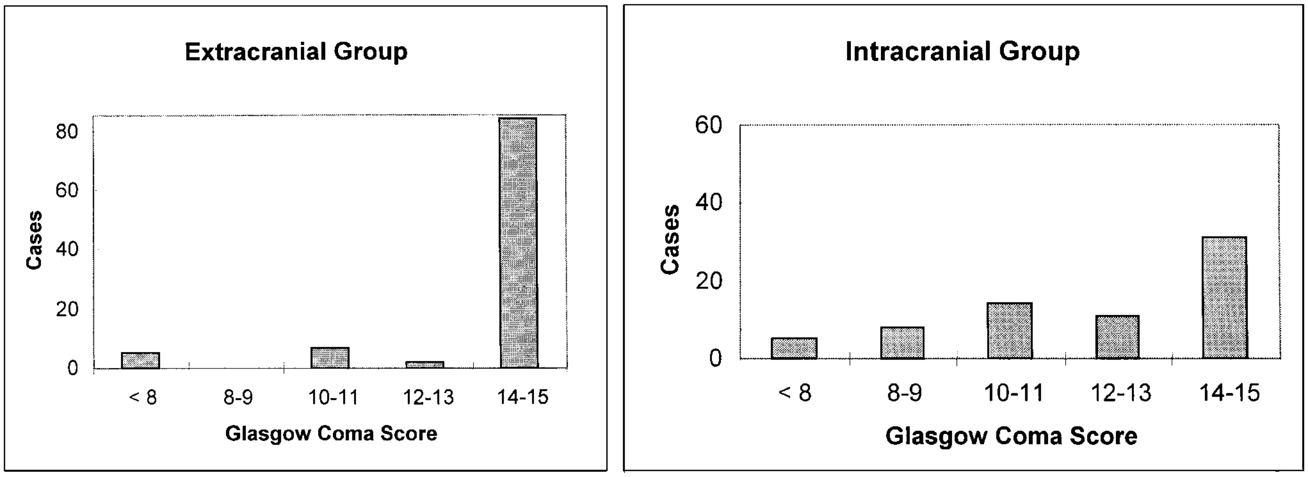


**Figure 2.** Comparison of the mental status of neurosurgery to facial surgery patients in the postanesthesia care unit. There are many more patients with low Glasgow Coma Scale scores in the intracranial surgery group (right) than in the extracranial group (left).

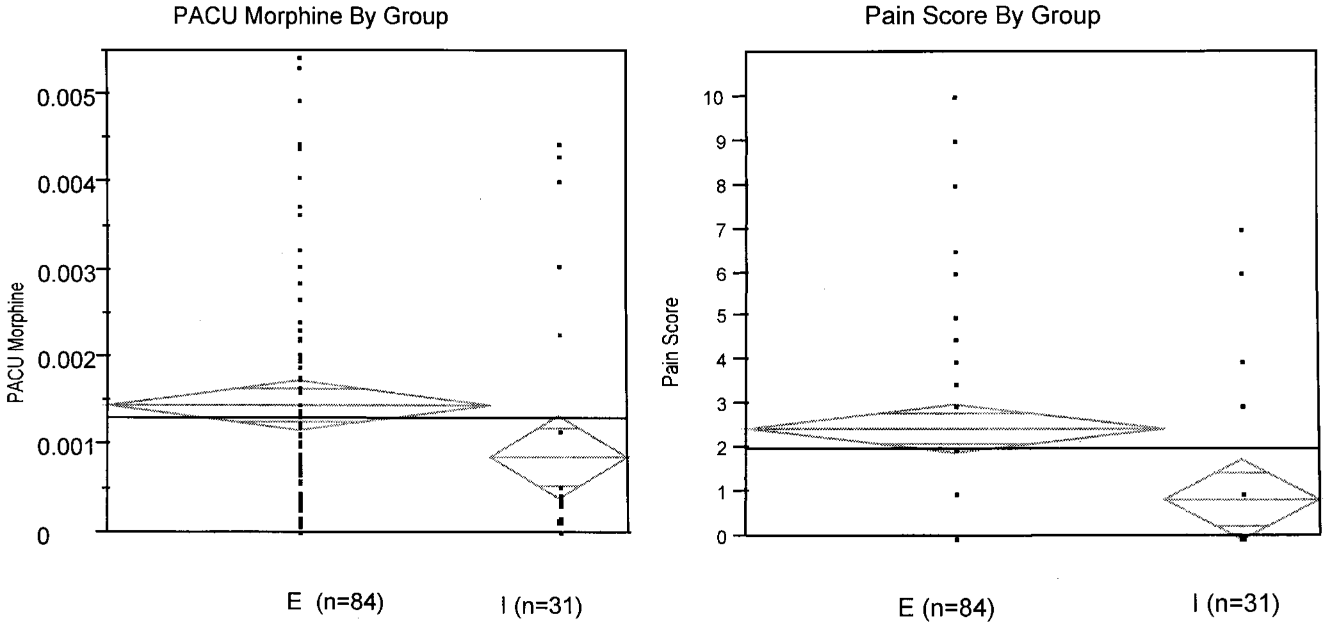


**Figure 3.** Morphine use (left) and pain reports (right) by patients in the postanesthesia care unit (PACU) who had Glasgow Coma Scale scores of 14 or 15 ($P < 0.001$). The difference in morphine use and pain report between the intracranial and extracranial surgery patients increases when mental status is removed as a covariant. The width of the diamonds is proportional to $n$ for each group. The mean is the line across the widest part of the diamond; the top and bottom of the diamonds represent 2 SD from the mean. The horizontal line across both plots indicates the population mean.

to severe postoperative pain. They suggest that postoperative pain in intracranial neurosurgical patients is undertreated by neurosurgeons for fear of either causing respiratory depression or interfering with neurological assessment, and that the observed differences in analgesic use between neurosurgical patients and other surgical patients was due to the reluctance of neurosurgeons to treat pain. However, De Benedittis et al. (1) did not account for intraoperative opioid use, nor did they compare the neurosurgical patients with a control group of nonneurosurgical patients.

Although there is no doubt that a significant number of patients in De Benedittis et al.'s series (1) experienced acute pain, it is difficult to put the magnitude of the pain in perspective without a control group. In a similar study, Quiney et al. (6) prospectively studied 52 patients after elective craniotomy. They reported that 18% of the patients complained of severe pain within 2 h of the surgical procedure and concluded that there was a need for reappraisal of pain relief after craniotomy. However, this study also lacks a control group, and, similarly, no mention is made of

**Table 2.** Morphine Use and GCS Scores for Craniotomy Patients Reporting Pain Scores >4

| PACU morphine (mg) | GCS score | Operative site |
|---|---|---|
| 21 | 14 | Transphenoidal hypophysectomy |
| 20 | 15 | Pterional craniotomy |
| 15 | 15 | Right subtemporal craniectomy |
| 13 | 15 | Left frontotemporal parietal craniotomy |
| 10 | 14 | Right frontotemporal parietal craniotomy |
| 10 | 15 | Transphenoidal hypophysectomy |
| 10 | 14 | Transphenoidal hypophysectomy |

GCS = Glasgow Coma Scale, PACU = postanesthesia care unit.

intraoperative opioid use. Moreover, despite the severe acute pain experienced by 18% of the patients, patients experienced very little pain after 12 h.

In a double-blinded study, Goldsack et al. (7) compared IM codeine with IM morphine for postoperative analgesia after intracranial surgery. They confirmed that morphine is a more potent and longer lasting analgesic than codeine. Acknowledging that the doses used in the study (10 mg of morphine versus 60 mg of codeine) were not equianalgesic (5), the authors nevertheless observed that none of the patients in the morphine group required more than two doses of morphine. Although this indicates that postoperative pain would be more appropriately treated with morphine than with codeine, their results also suggest that severe pain is not a persistent problem because only two doses of morphine were required. Again, without a control group and without consideration of intraoperative use of opioids, these results are suggestive but not conclusive.

In support of our observations, Williams et al. (8) reported that codeine 30–60 mg IM provides adequate pain relief in 90% of the postoperative craniotomy patients. This prospective study, which did not have a control group, showed that IM codeine, a drug that is variably absorbed (5) and classified as a weak opioid (9), nevertheless provides adequate analgesia in an overwhelming majority of neurosurgical patients undergoing craniotomy. Although the sample size is small, these results are consistent with the hypothesis that neurosurgical patients experience less pain than patients undergoing other surgical procedures.

To rule out systematic bias, our experimental design compared not only the use of analgesic between two different neurosurgical operative sites (Group I versus Group L), but also two anatomically proximated procedures (Group I versus Group E). The dissimilarity between Groups I and L suggests that it was not a surgical specialty-related bias and that the observed difference in pain scores and analgesia requirements between Groups I and E was real, although we cannot rule out the possibility that these two groups of patients were treated differently by the same surgeons. Perhaps more important is the fact that the culture in our institution is such that PACU nurses are instructed to administer opioids for adequate pain relief regardless of diagnosis or surgical procedure.

The gold standard for any clinical study is a prospective, randomized, double-blinded trial. To the extent that it is impossible to blind the surgical procedure, it may be difficult to completely eliminate investigator-associated bias in postoperative pain treatment. In this regard, the retrospective nature of the study has a unique advantage because there was no preconceived idea, and the nurses were not aware of the study or that patients who underwent craniotomy may have less intraoperative analgesic requirements. Nevertheless, the retrospective nature does have its limitations. The administrative pattern of intraoperative opioids was not tightly controlled. Although almost all patients received fentanyl intraoperatively, the method ranged from bolus administration to continuous infusion, as well as a combination of the two. In addition, the pharmacodynamic profile of different opioids could not be taken into account. Fortunately, as stated above, very few patients received an intraoperative opioid other than fentanyl. Concurrent medications were not controlled. All aneurysm patients received nimodipine prophylaxis for vasospasm and dilantin for potential seizures. The extent to which these drugs may affect analgesic requirements is unknown. However, patients with tumors or arteriovenous malformation did not receive these drugs, and they did not behave differently from patients with cerebral aneurysm. Finally, the data set is incomplete, and many patients had to be excluded. For example, of the 120 patients in the intracranial group, only 78 patients were admitted to the PACU and were therefore eligible for analysis. Theoretically, this could introduce a bias in that perhaps only patients who had a reduced or altered perception of pain had uneventful intracranial surgical procedures. Because there is no physiologic basis for this association, we reject this as a potential source of bias. Indeed, there is reason to believe that any bias should work against our hypothesis. With few exceptions, only patients who are neurologically intact at the end of an intracranial procedure are admitted to the PACU, and these patients should be able to appreciate pain. This selection bias may partially explain the observation that, as a group, the intracranial patients had a shorter stay in the PACU than the other patients. The more important explanation may, in fact, be the reduced expression of pain and, consequently, the reduced need for opioids. Because patients are not discharged from the PACU until postoperative pain is adequately controlled, and opioid administration may lead to respiratory depression and excessive sedation, patients in Groups E and L would have had a longer stay.

Our results are also consistent with those of earlier studies, in that some neurosurgical patients do experience severe pain and require potent analgesics, but not the same amount as other postoperative patients. This observation raises the following question: could

postoperative neurosurgical patients be unable to report their pain?

To remove the possibility that the patients' mental status affected their ability to express pain, leading to reduced administration of opioids, we reanalyzed a subset of data including only Group E and I patients with recorded GCS scores of 14 or 15. As with the larger data set, the difference in pain score and opioid use remained highly significant ($P < 0.0003$) (Figure 3).

Why did neurosurgical patients experience less pain less than other postoperative patients? Either they perceived a lesser nociceptive stimulus with surgical incision or they had an altered ability to experience pain; in effect, autoanalgesia. One factor contributing to the reduced nociception may be the site of surgery. The common neurosurgical incisions are in areas of reduced pain fiber density compared with incisions in the lumbar spine or the maxillofacial region (4). Moreover, dura, in contrast to the sinuses, is not richly innervated with pain receptors (4), and the brain itself is insensible to pain. These reasons may be sufficient to explain the reduced pain reported after intracranial neurosurgical procedures. Only patients who had a frontal craniotomy reported pain scores >4 or required >10 mg of morphine equivalents in the PACU (Table 2). De Benedittis et al. (1) also observed that subfrontal and temporal surgical approaches were associated with more severe pain.

Altered processing of nociception is the other hypothetical reason for reduced pain perception and consequent reduced opioid requirement. Archer and Samanani (10) showed that rats lesioned with a cryogenic insult but without observable neurological damage nevertheless had reduced tail-flick latency 72 h after injury. The authors concluded that there was an alteration of nociception and suggested that the injury had activated the bulbospinal serotonergic pathways. These observations, as well as those from an earlier study by the same authors (11), suggest a possible mechanism for our findings. Facilitation of afferent inhibitory activity, both at the level of the dorsal horn and at the level of the hippocampus or the amygdala, would reduce the subjective experience and, presumably, report of the pain sensation. It is obvious that observations made in the rat cannot be applied directly to humans, and that brain retraction or surgical trauma is not equivalent to cryogenic injury. Furthermore, if this hypothesis explains the reduced pain perception after neurosurgery, then the autoanalgesic effect may persist for days after surgery. Nevertheless, this provides a plausible mechanism for the observed results.

Further study is required to elucidate a mechanism to account for the observed results. Regardless of the mechanism, we have established that, after intracranial surgery, patients have reduced analgesic requirements compared with other patients who have undergone invasive surgical procedures. Furthermore, we have identified a subgroup of patients who may have greater postoperative analgesic requirements than others—patients who have undergone a frontal craniotomy.

In conclusion, our data support the notion that patients do experience less pain after craniotomy procedures compared with other procedures. Although individual patients may experience severe pain, the general approach of judicious administration of opioids remains valid.

The authors gratefully acknowledge Paula Pedersen and Karen Rutherford for their assistance in the preparation of this manuscript.

## References


1. De Benedittis G, Lorenzetti A, Migliore M, et al. Postoperative pain in neurosurgery: a pilot study in brain surgery. Neurosurgery 1996;38:466–70.
2. Stoneham MD, Walters FJ. Post-operative analgesia for craniotomy patients: current attitudes among neuroanaesthetists. Eur J Anaesthesiol 1995;12:571–5.
3. Stoneham MD, Cooper R, Quiney NF, Walters FJ. Pain following craniotomy: a preliminary study comparing PCA morphine with intramuscular codeine phosphate. Anaesthesia 1996;51:1176–8.
4. Bonica JJ. The management of pain. Vol 1, 2nd ed. Philadelphia: Lea & Febiger, 1990.
5. United States Department of Health and Human Services. Acute pain management: operative or medical procedures and trauma. In: Clinical practice guideline. Washington, DC: United States Department of Health and Human Services, Agency for Health Care Policy and Research, 1992.
6. Quiney N, Cooper R, Stoneham M, Walters F. Pain after craniotomy: a time for reappraisal? Br J Neurosurg 1996;10:295–9.
7. Goldsack C, Scuplak SM, Smith M. A double-blind comparison of codeine and morphine for postoperative analgesia following intracranial surgery. Anaesthesia 1996;51:1029–32.
8. Williams JM, Craen RA, Novick T, Koman W. The efficacy of intramuscular (IM) codeine for post-craniotomy pain [abstract]. Can J Anaesth 1997;44:A28B.
9. United States Department of Health and Human Services. Management of cancer pain. In: Clinical practice guideline. Washington, DC: United States Department of Health and Human Services, Agency for Health Care Policy and Research, 1994.
10. Archer DP, Samanani N. The influence of cryogenic brain injury on nociception in the rat. Anesthesiology 1993;78:937–44.
11. Archer DP, Priddy RE, Tang TK, et al. The influence of cryogenic brain injury on the pharmacodynamics of pentobarbital: evidence for a serotonergic mechanism. Anesthesiology 1991;75:634–9.
