# Supplementary material for: Postoperative analgesia of scalp nerve block with ropivacaine in pediatric craniotomy patients: a protocol for a prospective, randomized, placebo-controlled, double-blinded trial
Source: Trials. 2020 Jun 26;21:580. doi: 10.1186/s13063-020-04524-7 (PMC7318534; doi:10.1186/s13063-020-04524-7)
Supplement: Supplementary file 1 — Additional file 1. [file 13063_2020_4524_MOESM1_ESM.zip › reference 8R2.pdf]

Suboptimal pain treatment after craniotomy

Morten Sejer Hansen[1], Jannick Brennum[2], Finn Borgbjerg Moltke[3] & Jørgen Berg Dahl[1]

ABSTRACT

INTRODUCTION: Only few studies have investigated pain, nausea, sedation and analgesic strategies in post-craniotomy patients. The aim of this observational study was to explore pain, nausea, sedation and analgesic procedures after craniotomy, and to evaluate the quality of current analgesic therapy administered to post-craniotomy patients.

MATERIAL AND METHODS: A total of 59 patients undergoing supratentorial or infratentorial craniotomy were included over a three-month period. The intensity of pain, nausea and sedation was evaluated at one, two, four, eight and 24 h after extubation. Post-operative analgesic consumption at 0-48 h after extubation was noted. Post-operative morphine consumption in relation to gender, surgical procedure, administration of preoperative steroids and application of surgical drains was evaluated.

RESULTS: Fifty patients completed the study. After the first post-operative hour, 56% suffered from moderate-to-severe pain, which decreased to 38% at 24 h post-operatively. Patients receiving preoperative steroids experienced significantly less pain than patients who did not receive preoperative steroids ($p = 0.04$). The mean post-operative morphine consumption 0-48 post-operatively was 28.8 mg (± 23.6 mg). Only 52% of the patients received the planned amount of acetaminophen of 4,000 mg/day.

CONCLUSION: Pain following craniotomy is moderate to severe in a substantial number of patients. The quality of the analgesic treatment leaves room for improvement. Administration of preoperative steroids may reduce post-craniotomy pain.

FUNDING: not relevant.

TRIAL REGISTRATION: not relevant.

In 1996, De Benedittis et al performed a pilot study demonstrating that 60% of post-craniotomy patients suffered from moderate-to-severe post-operative pain [1]. Prospective observational studies have subsequently confirmed these findings [2-5]. However, despite the growing interest in post-craniotomy pain, a gold standard for analgesic therapy in these patients is still lacking [6, 7]. An optimal analgesic therapy is important, considered the fact that sub-optimal postoperative analgesia is not only of discomfort to the patient, but may also lead to an increase in the incidence of post-operative complications, prolonged hospital stay and, in turn, to an increase in health expenses [8]. Additionally, intense post-operative pain following craniotomy can result in sympathetically induced arterial hypertension, which increases the risk of developing secondary intracranial haemorrhage [9].

The aim of this prospective observational study was to explore pain intensity, nausea, sedation and analgesic management after craniotomy. In particular, we hypothesized that administration of preoperative steroids, supratentorial surgery, avoidance of surgical drains and male gender would be associated with reduced post-operative pain scores. Furthermore, we wanted to evaluate the current analgesic therapy administered to the post-craniotomy patients.

MATERIAL AND METHODS

This prospective observational study was carried out at the Department of Neurosurgery, Rigshospitalet, Denmark. Data from 59 patients undergoing elective supratentorial or infratentorial craniotomy in the period from November 2010 to January 2011 were collected. The local research ethics committee was informed, but deemed it unnecessary to consider the study for approval due to its strictly observational character.

Informed consent was, however, obtained from all patients.

The inclusion criteria were patients over the age of 18 years who presented for elective craniotomy at Rigshospitalet, were able to speak and understand Danish, did not have a history of substance abuse and were not diagnosed with chronic pain. The exclusion criteria were age under 18 years, difficulties in communication, Burr-hole trephination, shunt implantation, emergency surgery, brain biopsies, transphenoidal surgery of the pituitary gland and extubation later than 1 h after the end of surgery.

On the day before surgery, patients were interviewed and received information regarding perioperative analgesic and antiemetic use. Furthermore, they were instructed in the use of the 11-point numeric rating scale (NRS) for pain assessment, and a four-point nausea and sedation score (0: no nausea/sedation, 1: light nausea/sedation, 2: moderate nausea/sedation, 3: severe nausea/sedation). After instruction patients were scored for NRS and nausea at rest.

Standard anaesthetic technique was used: Induction with propofol 2 mg/kg, followed by maintenance of anaesthesia with remifentanil 30 microgram/kg/h and propofol 5 mg/kg/h. All patients received 0.1

ORIGINAL ARTICLE

1) Department of Anaesthesia 4231, Centre of Head and Orthopaedics, Rigshospitalet
2) Department of Neurosurgery, Rigshospitalet
3) Department of Neuroanaesthesia, Rigshospitalet

Dan Med J 2013;60(2):A4569

TABLE 1

Data on included patients.

Male/female, n	23/27
Age, mean (± standard deviation), years	55 (± 13.2)
Type of craniotomy, n	
Tumour cerebri	24
Tumour cerebri meningeoma	7
Trigeminal neuralgia	2
Skull base	7
Posterior fossa tumour	5
Aneurysm	1
Acoustic neuroma	2
Cerebral arteriovenous malformation	1
Pituitary tumour	1
Surgical site, n	
Supratentorial	29
Infratentorial	
Application of surgical drain, n	31
Administration of preoperative steroids, n	20

TABLE 2

Patients receiving acetaminophen in the first 48 post-operative hours.

Dose, mg	Patients, n
8,000	26
7,000	12
6,000	3
5,000	5
4,000	2
1,000	1

mg/kg intravenous morphine 30 minutes before wound closure.

Data regarding surgical procedure (supratentorial versus infratentorial), anaesthetic, and administered analgesics and antiemetics were documented. Post-operative pain, nausea and sedation were recorded at one, two, four, eight, and 24 h after extubation. Post-operative analgesic and antiemetic consumption was recorded 48 h post-operatively. For comparison, post-operative opioids were converted into morphine equivalents. All patients were prescribed standard analgesic treatment consisting of oral acetaminophen 4,000 mg/day with supplemental morphine (intravenous or oral) as needed.

Trial registration: not relevant.

Statistics

All statistical analyses were performed with SPSS for Windows, version 18.0.

Mean and median values and standard deviations of post-operative NRS score, nausea and sedation scores were calculated. We calculated the mean value of post-operative morphine consumption 48 h post-operatively and the area under the curve (AUC) of NRS scores from patients stratified according to gender and to administration of preoperative steroids versus no preoperative steroids.

The Mann-Whitney U test was used to compare NRS scores, nausea, and sedation scores, and post-operative morphine consumption between genders along with type of surgical procedure, administration of preoperative steroids and application of surgical drains.

RESULTS

Fifty-nine patients were included in the study. A total of nine patients were excluded. Five patients were excluded due to cancellation of surgery, one was intubated 8 h post-operatively and three patients developed aphasia. In ten patients, data on post-operative pain, nausea, vomiting and sedation were incomplete (i.e. measurements were not made at all five time points). The collected data from these patients were included in the analysis. Data of the included patients are presented in **Table 1**.

After the first post-operative hour, 56% of the included patients had moderate to severe pain (NRS 4-10), which decreased to 38% at 24 h post-operatively. The pain intensity during the observational period is illustrated in **Figure 1**. The median NRS score during the 24-h study period ranged from two to four.

Patients receiving preoperative steroids experienced significantly less pain than patients receiving no steroids ($p = 0.04$) (**Figure 2**). No significant differences in pain score or post-operative analgesic consumption were observed when comparing surgical site (infratentorial versus supratentorial), application of surgical drains, or gender. However, we observed a tendency towards higher pain scores in females and in patients undergoing infratentorial surgery.

The incidence of post-operative nausea and vomiting was low with 86% of the patients experiencing no nausea after the first post-operative hour and 78% experiencing no pain at 24 h. Likewise, the incidence of sedation in the first 24 post-operative hours was low as 62% of the patients experienced no sedation and 22% experienced only light sedation at 24 h post-operatively. The mean post-operative morphine consumption in the first 48 h post-operatively was 28.8mg (± 23.6 mg.). Only 52% of the patients received the planned 4,000 mg/day of acetaminophen (**Table 2**).

DISCUSSION

The study demonstrated that 56% of the patients had moderate-to-severe pain after the first post-operative hour with a median NRS score of four. Furthermore, the

study demonstrated significant differences in post-operative pain when NRS scores from patients receiving preoperative steroids were compared with scores from patients who did not receive preoperative steroids. A rather low incidence of nausea, vomiting and sedation was demonstrated. Finally, only 52% of the patients received the planned 4,000 mg/day of acetaminophen (Table 2).

Insufficient analgesic treatment causes discomfort to the patient and may also lead to an increase in the incidence of post-operative complications. In the post-craniotomy patient, post-operative pain resulting in an increase in arterial blood pressure may increase the risk of intracranial haemorrhage [9]. The rather high number of patients suffering from moderate-to-severe pain during the first 24 post-operative hours demonstrates the need for an improved analgesic strategy in post-craniotomy patients.

Steroids have a well-known effect on post-operative nausea and vomiting (PONV) and pain [10]. Mordhorst et al demonstrated that use of intraoperative steroids reduced the risk of post-craniotomy pain [5]. In accordance with our hypothesis, administration of preoperative steroids to patients mainly with malignancies was associated with significantly lower pain scores up to 24 h post-operatively (Figure 2). These observational findings are encouraging, but have to be documented together with the potential side effects in further randomised controlled trials.

In contrast to our initial hypothesis, no significant differences in pain scores were demonstrated in this study when comparing gender, type of surgery and application of surgical drains. Several studies have demonstrated significant differences in pain scores when comparing gender and surgical procedure. However, the insignificant result in this trial was most probably due to the rather low number of patients included.

The mean cumulated post-operative morphine consumption of 28.80 mg (± 23.6 mg) 0-48 h post-operatively may seem low considering that a rather large number of patients suffered from moderate-to-severe pain. The use of opioids in post-craniotomy patients is controversial. Their use in this particular setting may be limited by the potential risk of respiratory depression, sedation, nausea and vomiting, and by the fact that opioids may mask neurological assessment. As discussed in a recent review [7], only one double-blinded, placebo-controlled randomised trial (RCT) investigating morphine has been performed in this setting [11]. The trial demonstrated no significant opioid-related side-effects of patient-controlled morphine (PCA-morphine).

In our department, the basic analgesic regimen in post-craniotomy patients consists of acetaminophen 4,000 mg/day with supplemental intravenous or oral morphine as needed. Fear of opioid-related side-effects combined with insufficient amounts of acetaminophen means that the risk of moderate-to-severe post-operative pain is imminent. The reason why only 52% of the patients received the prescribed amount of acetaminophen of 4,000 mg/day remains unclear, but similar results were recently documented by other departments at our institution [12].

In addition to pain, PONV is a major concern after craniotomy. PONV is a serious complication that can lead to a potential elevation of intracranial pressure which may, in turn, increase the risk of haemorrhage.

FIGURE 1

Post-operative numeric rating scale score 0-24 h.

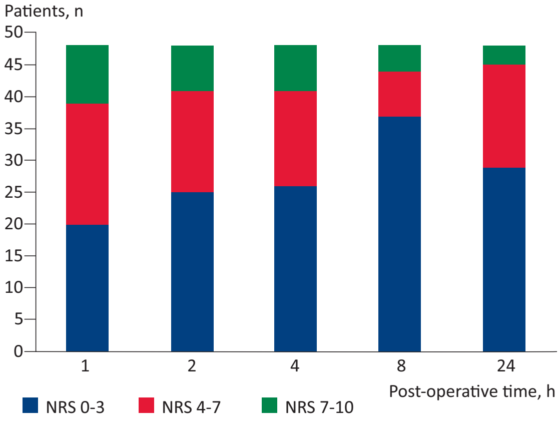

NRS = numeric rating scale

FIGURE 2

Comparison of the percentage of patients with a numeric rating scale score of 4-10 receiving preoperative steroids versus no preoperative steroids. p = 0.04 (area under the curve) between groups.

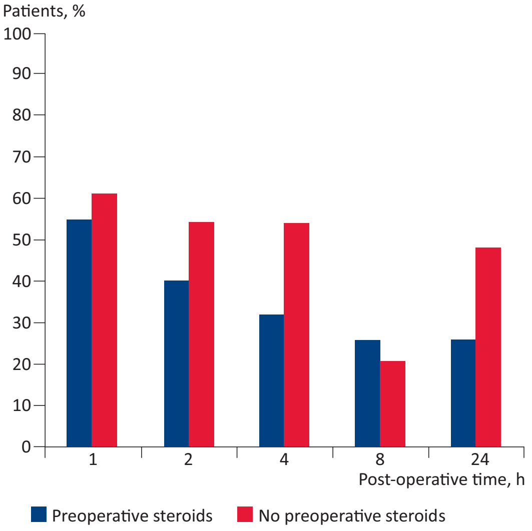

NRS = numeric rating scale

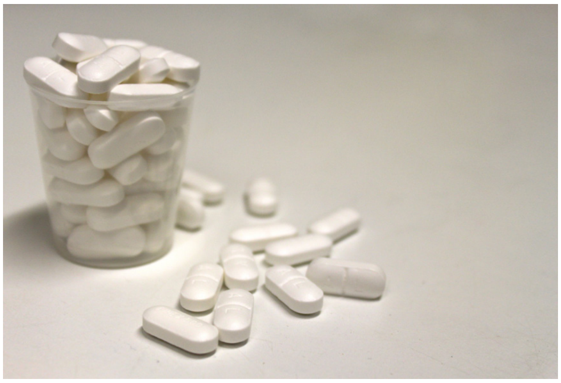

Pain treatment after craniotomy. A new approach is needed.

Contrary to our initial hypothesis, the incidence of PONV was rather low in this study. The limited use of morphine and successful antiemetic strategies may have contributed to this. Administration of preoperative steroids with a known antiemetic effect had no significant effect on nausea in our rather small observational study, but this result should not be considered conclusive.

In conclusion, 56% of the patients included in this descriptive study experienced moderate to severe pain during the first post-operative hours, and 38% at 24 h post-operatively. A rather low incidence of nausea, vomiting and sedation was documented. Only 52% of the patients received the prescribed amount of acetaminophen in the first 48 h. Our observations indicate that administration of preoperative steroids may reduce post-craniotomy pain. Our study thus adds to the evidence that post-craniotomy pain as well as its handling seems to be a significant problem which leaves room for improvement. In addition, there is an urgent need for well-performed RCTs on pain therapy following craniotomy [7].

CORRESPONDENCE: *Morten Sejer Hansen,* Anæstesiologisk Klinik 4231, HovedOrtoCentret, Rigshospitalet, 2100 Copenhagen, Denmark.
E-mail: morten_sejer@yahoo.dk

ACCEPTED: 14 November 2012

CONFLICTS OF INTEREST: None. Disclosure forms provided by the authors are available with the full text of this article at www.danmedj.dk.

ACKNOWLEDGMENTS: We hereby extend our gratitude to the nursing personnel at Department of Neurosurgery, Neurosurgical Operating Theatre, and the Post Anaesthesia Care Unit, Rigshospitalet, for collection of data.

LITERATURE

1. De Benedittis G, Lorenzetti A, Migliore M et al. Postoperative pain in neurosurgery: a pilot study in brain surgery. Neurosurgery 1996;38:466-9,discussion 9-70.
2. Klimek M, Ubben JF, Ammann J et al. Pain in neurosurgically treated patients: a prospective observational study. J Neurosurg 2006;104:350-9.
3. Gottschalk A, Berkow LC, Stevens RD et al. Prospective evaluation of pain and analgesic use following major elective intracranial surgery. J Neurosurg 2007;106:210-6.
4. Quiney N, Cooper R, Stoneham M et al. Pain after craniotomy. A time for reappraisal? Br J Neurosurg 1996;10:295-9.
5 . Mordhorst C, Latz B, Kerz T et al. Prospective assessment of postoperative pain after craniotomy. J Neurosurg Anesthesiol 2010;22:202-6.
6. Nemergut EC, Durieux ME, Missaghi NB et al. Pain management after craniotomy. Best Pract Res Clin Anaesthesiol 2007;21:557-73.
7. Hansen MS, Brennum J, Moltke FB et al. Pain treatment after craniotomy: where is the (procedure-specific) evidence? A qualitative systematic review. Eur J Anaesthesiol 2011;28:821-9.
8. Leslie K, Troedel S, Irwin K et al. Quality of recovery from anesthesia in neurosurgical patients. Anesthesiology 2003;99:1158-65.
9. Basali A, Mascha EJ, Kalfas I et al. Relation between perioperative hypertension and intracranial hemorrhage after craniotomy. Anesthesiology 2000;93:48-54.
10. Hockey B, Leslie K, Williams D. Dexamethasone for intracranial neurosurgery and anaesthesia. J Clin Neurosci 2009;16:1389-93.
11. Jellish WS, Leonetti JP, Sawicki K et al. Morphine/ondansetron PCA for postoperative pain, nausea, and vomiting after skull base surgery. Otolaryngol Head Neck Surg 2006;135:175-81.
12. Mathiesen O, Thomsen BA, Kitter B et al. Need for improved treatment of postoperative pain. Dan Med J 2012;59(4):A4401.
